# Grown to Be Blue—Antioxidant Properties and Health Effects of Colored Vegetables. Part II: Leafy, Fruit, and Other Vegetables

**DOI:** 10.3390/antiox9020097

**Published:** 2020-01-23

**Authors:** Francesco Di Gioia, Nikolaos Tzortzakis, Youssef Rouphael, Marios C. Kyriacou, Shirley L. Sampaio, Isabel C.F.R. Ferreira, Spyridon A. Petropoulos

**Affiliations:** 1 Department of Plant Science, The Pennsylvania State University, University Park, PA 16802, USA; fxd92@psu.edu; 2 Department of Agricultural Sciences, Biotechnology and Food Science, Cyprus University of Technology, 3603 Lemesos, Cyprus; nikolaos.tzortzakis@cut.ac.cy; 3 Department of Agricultural Sciences, University of Naples Federico II, 80055 Portici, Italy; youssef.rouphael@unina.it; 4 Department of Vegetable Crops, Agricultural Research Institute, 1516 Nicosia, Cyprus; m.kyriacou@ari.gov.cy; 5 Centro de Investigação de Montanha (CIMO), Instituto Politécnico de Bragança, Campus de Santa Apolónia, 5300-253 Bragança, Portugal; shirleylsampaio@gmail.com (S.L.S.);; 6 Department of Agriculture, Crop Production and Rural Environment, University of Thessaly, 38446 N. Ionia, Magnissia, Greece

**Keywords:** anthocyanins, antioxidants, flavonoids, fruit vegetables, functional quality, leafy vegetables, inflorescence, lettuce, natural colorants, tomato

## Abstract

The current trend for substituting synthetic compounds with natural ones in the design and production of functional and healthy foods has increased the research interest about natural colorants. Although coloring agents from plant origin are already used in the food and beverage industry, the market and consumer demands for novel and diverse food products are increasing and new plant sources are explored. Fresh vegetables are considered a good source of such compounds, especially when considering the great color diversity that exists among the various species or even the cultivars within the same species. In the present review we aim to present the most common species of colored vegetables, focusing on leafy and fruit vegetables, as well as on vegetables where other plant parts are commercially used, with special attention to blue color. The compounds that are responsible for the uncommon colors will be also presented and their beneficial health effects and antioxidant properties will be unraveled.

## 1. Introduction

Vegetables are considered an invaluable ingredient of human diet, since they diversify color of various food products and they also possess beneficial health effects due to their content in various phytochemicals such as flavonoids, betalains and carotenoids and the overall high antioxidant capacity [1,2,3]. Consumption of purple/blue fresh produce is associated with increased nutrient intake and reduced risk for metabolic syndrome [4]. Based on food intake data from NHANES 2001–2002, the daily intake of anthocyanins was estimated to be 12.5 mg/day/person in the United States [5]. The predominant dietary anthocyanins are malvidin, delphinidin, and peonidin glycosides [6], which can be found in many plant foods, including berries, purple sweet potatoes, grapes, and wine [7]. In comparison with other flavonoids, anthocyanins possess a positive charge on their C-ring, which leads to different colors in response to various pH [8].

Anthocyanins not only have aesthetic importance by generating characteristic purple, bluish, orange, and reddish pigments in various plant tissues [9], but also have biological functions including protective effects against radiation, reactive oxygen species scavenging, defense against pathogens and stress conditions, and attracting seed and pollen dispersers [10,11,12]. These compounds also have nutritional value and potential health benefits [13].

Leafy vegetables are widely consumed throughout the world and they significantly contribute to the overall recommended daily intake for several nutrients essential for human body [14]. They include several species among which lettuce is considered the most important salad vegetable and several reports highlighted its significance in human nutrition [15,16]. On the other hand, the Solanaceae family has approximately 2700 species and 99 genera and includes some of the most important fruit vegetables consumed globally. *Solanum*, the largest and most complex genus in this family, is of great economic importance with several species used as foods, medicines, and ornamental plants [17]. In other popular vegetables consumed worldwide such as broccoli, cauliflower, and artichoke, the immature inflorescence constitutes the edible portion and represent a rich source of flavonoids, anthocyanins, and other bioactive compounds that are also responsible for their pigmentation [18,19,20,21]. In the case of sweet corn, another popular vegetable used for fresh consumption as well as for canned and freezing processing, differing from the regular corn only for a higher accumulation of sugar in the kernels, the ear including cob and kernels constitute a rich source of natural colorants including carotenoids and flavonoids [22]. Yet, in other cases like asparagus the edible portion and source of anthocyanins is constituted by the young stems [23].

Recently we published a review paper regarding colored root vegetable species focusing on the most important coloring compounds and their antioxidant effects. With the present review we aim to continue this work and present the rest of colored vegetable crops, focusing on leafy and fruit vegetables and relevant species where other plant parts are consumed. Having in mind the same context, the main coloring compounds are highlighted, while a special section for each species is allocated to their health effects and antioxidant properties. The information presented in this review was systematically gathered from scientific databases such as Scopus, ScienceDirect, PubMed, Google Scholar, and ResearchGate by using various keywords and key phrases, e.g., the common and Latin of the main species and/or the terms “health effects”, “antioxidant compounds”, “colored leafy vegetables”, “colored fruit vegetables”, “blue vegetables”, “purple vegetables”, and “anthocyanins”.

## 2. Leafy Vegetables

### 2.1. Lettuce

Lettuce (*Lactuca sativa* L.) belongs to the Asteraceae (Compositae) family and is a very popular vegetable crop used for fresh consumption and as salad ingredient owing to its sensory and health-promoting properties [14,24,25,26]. Lettuce is widely cultivated throughout the globe and it is rightly considered the most important of leafy greens as it ranks highest in production (27 million tons in 2017; [27]). Lettuce comes in a wide variety of head formations, textures, sizes, leaf shapes, and colors and it is conventionally classified according to Mou [28] into six major groups (i.e., types): (i) Butterhead, (ii) Cos or Romaine, (iii) Crisphead, (iv) Leaf or Cutting, v) Stalk or Stem, and vi) Latin. Compared to several other leafy vegetables, lettuce is an excellent source of vitamin B9 and total flavonoids. Wang et al. [29] and Gan and Azrina [30] reported that total vitamin B9 and flavonoid contents in lettuce were higher by 16% and 220%, respectively, compared to spinach, which is another important and widely consumed leafy green. Quali-quantitative variation in lettuce vitamins and secondary metabolites (i.e., phytochemicals) depends on many pre-harvest factors such as genotype, environmental conditions, harvest maturity, and agricultural practices [31]. However, the genetic material is the predominant pre-harvest factor and the major determinant of the biosynthesis and accumulation of lipophilic (i.e., carotenoids, chlorophylls, and vitamin E) and hydrophilic (i.e., phenolic compounds and vitamin C) antioxidant molecules [32]. Vitamins are essential micronutrients required for human metabolism and functionalities implicated in the reduction of cardiovascular and degenerative diseases [33]. Folate (vitamin B9) and vitamin C (as ascorbic and dehydroascorbic acids) are eminently present in lettuce [14]. In their review article Kim and co-workers reported that folate and vitamin C contents vary with leaf type and particularly leaf coloration, with red leaf, butterhead, and romaine lettuces being particularly good sources of folate [15,29,34], while leaf green lettuce had the highest vitamin C concentration [24,25,34,35].

Carotenoids, which constitute an important group of lipophilic pigments frequently present in yellow-orange vegetables and in dark green leafy vegetables, vary in concentration among lettuce types and colors. Mou [36] assessed the genetic variability in β-carotene and lutein content, the most abundant carotenoids in lettuce, across 52 genotypes (including butterhead, crisphead, Latin, leaf, primitive, romaine, stem lettuce and wild species) that were categorized by type in the following order: Romaine and green leaf > red leaf > butterhead > crisphead. The author also reported that the two target carotenoids were significantly and positively correlated with chlorophyll a and b as well as with total chlorophyll content. Contrarily to the findings of Mou [36], Baslam et al. [37], and Nicolle et al. [25] demonstrated that the content of β-carotene and lutein may not entirely correlate with leaf green pigmentation, since the carotenoids content appeared to be lower in green compared to red-pigmented lettuce plants. The contradiction between these results may indicate that the content in carotenoids may not be consistently related to leaf pigmentation [14]. Nevertheless, the frequent consumption of carotenoids-rich lettuce could be of high importance since several epidemiological studies demonstrated that the onset of chronic diseases such as heart disease, vision impairment, and certain types of cancer (lung, prostate, and colon) could be reduced [38,39,40,41].

Several authors reported that the contents of secondary metabolites in lettuce differed greatly among genotypes and depended particularly on leaf color (dark red, red, green/red, and green) [14,42]. According to Mulabagal et al. [43] red lettuce contains a single anthocyanin, namely cyanidin-3-*O*-(6’’-malonyl-β-glucopyranoside) which is further converted in two cyanidin derivatives (cyanidin-3-*O*-(6’’-malonyl-β-glucopyranoside methyl ester) and cyanidin-3-*O*-β-glucopyranoside), all presenting significant antioxidant activities against lipid peroxidation and cyclooxygenase activity. Kim et al. [42] explored the genetic material of 23 lettuce cultivars belonging to three major lettuce groups (crisphead, oak-leaf, and romaine) in respect to their phytochemical content and antioxidant potential. The authors reported that most phytochemicals varied significantly with genetic material and were associated mainly with leaf color. The red-leaf and to a lesser extent the green/red cultivars exhibited the highest concentration of the following antioxidant molecules: Cyanidin, carotenoids (lutein, violaxanthin and lactucaxanthin), fatty acids such as α-linolenic and linoleic acid, total polyphenols, and antioxidant potential [42]. In the same study, the authors were also able to demonstrate that the methanolic extract of red-pigmented lettuce contained potent scavengers of ABTS (scavenging assay of 2,2-azino-bis(3-ethylbenzthiazoline-6-sulphonic acid) diammonium salt radical) and DPPH (scavenging assay of 2,2-diphenyl-1-picrylhydrazyl radical) radicals. The higher radical-quenching activity of red-pigmented lettuce cultivars irrespective of type renders them more bioactive thus their systematic inclusion in the human diet could be an efficient tool to minimize the impact of oxidative stress-related diseases [25]. Similarly, Hao et al. [44], reported the differences in nutritive quality (e.g., cellulose, protein, starch, sugar, and vitamin C contents) among 74 red/purple and green varieties of leaf lettuce using grey correlation analysis (a methodology of treating and analyzing qualitative data according to grey system procedure). The authors concluded that purple-leaf lettuce “P-S23’’ exhibited significantly higher grey comprehensive evaluation value (0.8) in comparison to the green counterparts (values < 0.5). In addition, leaf pigmentation significantly correlates to the constitution and concentration of phenolic compounds belonging mainly in the subgroups of phenolic acids, flavonoids, and anthocyanins [14]. Chicoric, caffeic, and chlorogenic acids and their derivatives are the most abundant phenolic acids present in lettuce, whereas the most outstanding flavonoids include anthocyanins, quercetin, kaempferol, and flavone luteolin [35,45,46,47]. Several authors reported a higher total phenolic content in red butterhead, red leaf and red romaine lettuces compared to their green counterparts [16,35,48,49,50,51]. The red color of lettuce has been associated with a higher total phenolics content, known to impart a greater antioxidant activity than vitamins C and E [52], and has been attributed primarily to anthocyanins, an important group of flavonoids responsible for the red/purple coloration [53].

Regarding the health benefits attributed to lettuce, according to an in vivo study carried out by Lee et al. [54] on mice fed with a high-fat diet, supplementation of the diet with 8% red lettuce on a body weight (bw) basis decreased the total cholesterol and the low density lipoprotein (LDL) by 9% and 123%, respectively, thus highlighting the potential effects of red-pigmented lettuce consumption against cardiovascular disease. A putative mechanism behind the cholesterol reduction could be the synergistic effects of lipophilic and hydrophilic antioxidant molecules such as α-tocopherol, anthocyanins, β-carotene, and phenolic compounds. Similarly, Nicolle et al. [24] observed that feeding male rats with 20% of red oak-leaf lettuce decreased significantly the LDL (low density lipoproteins)/HDL (high-density lipoproteins) cholesterol ratio and the liver cholesterol content. 

In addition to preclinical trials, clinical studies demonstrated that weekly consumption of lettuce was able to reduce the incidence of colorectal cancer [55]. The protective effect of frequent lettuce consumption against colorectal cancer has been attributed to the presence of β-carotene and vitamin C and not to Ca and vitamins B9 and E contents [55]. Recently, Qin [56] reported that the new cultivar B-2 of red-pigmented lettuce, characterized by high concentration of anthocyanins, flavones, and phenolic acids, can minimize oxidative stress-related diseases, leading to anti-tumor effects against human lung adenocarcinoma, hepatoma, and human cancer colorectal adenoma cell lines. Based on the above considerations, clinical and preclinical studies demonstrated that frequent consumption of fresh lettuce, in particular the red-pigmented varieties, carries potential anti-diabetic, cholesterol lowering, and anti-tumor properties.

### 2.2. Basil

Basil (*Ocimum basilicum* L.) belongs to the Lamiaceae family and it is one of the most important aromatic herbs cultivated worldwide as it flourishes under a wide range of climatic conditions. Basil cultivated both in open-field and under greenhouse conditions is an essential ingredient of renowned pesto sauce (widely consumed in Italy), while it is also used for fresh consumption and as a culinary spice [57]. In addition, the extraction of essential oils from basil is of high interest to both cosmetic and pharmaceutical industries. Basil comes in a wide variety of types, conventionally classified into seven morphotypes: (i) Large-leafed “Italian” basil, (ii) tall, slender basil, (iii) dwarf “Bush” basil, (iv) compact “Thai” basil, (v) purple basil (with clove-like aroma), (vi) citriodorum basil (flavored types), and (vii) purpurascens basil (sweet purple-colored basil) [58]. The herbs of the Lamiaceae family, such as basil, are characterized by strong antioxidant capacity. Basil in particular is a rich source of phenolic compounds, including phenolic acids such as rosmarinic, caffeic, chicoric and caftaric acids [59,60,61], vitamin C, and carotenoids such as lutein and β-carotene [62]. Furthermore, certain purple/red cultivars also have important concentrations of the hydrophilic anthocyanins, especially the “Dark Opal”, “Purple Ruffles”, and “Rubin” cultivars” [61,63].

The main carotenoids detected in basil are mostly lutein and β-carotene; but Calucci et al. [64] ranks basil first among aromatic herbs with respect to the concentrations of xanthophyll carotenoids. In the study of Kopsell et al. [62], the main detected carotenoids in sweet basil were identified as lutein, β-carotene, and zeaxanthin, and significant differences in carotenoid profiles were observed between different growing conditions (open-field versus greenhouse) and cultivars (“Cinnamon”, “Genovese”, “Italian large leaf”, “Nufar”, “Osmin purple”, “Red Rubin”, “Spicy bush”, and “Sweet Tai”). According to Marchand et al. [38] and Johnson et al. [40], the frequent consumption of vegetables and herbs was more strongly correlated with reduced risk of certain types of cancer and degenerative ophthalmic diseases, in comparison to the ingestion of monomolecular carotenoid supplements. In addition to carotenoids, basil is considered also an important source of vitamin C. It is well established that vitamin C is crucial for immune and antioxidant functions, and according to the WHO, 80–90 mg of vitamin C should be ingested daily [65]. However, due to the water solubility of ascorbic acid, regular dietary intake is essential to normal metabolic functioning [66]. According to Murarikova and Neugebauerova [65] the ascorbic acid content varied from 34.3 to 220.0 mg/kg fresh weight among the tested varieties (“Dark Green”, “Lettuce Leaf”, “Mammolo Genovese”, “Manes”, “Ohre”, “Purple Opal”, and “Red Rubin”) and the different growing seasons.

Rosmarinic acid is noted in the scientific literature as the most abundant phenolic constituent of basil [59,60]. On the other hand, a study carried out by Kwee and Niemeyer [61] showed that 9 over 15 basil cultivars contained other caffeic acid derivatives, such as chicoric acid, in higher concentrations than rosmarinic acid. Moreover, the antioxidant and antimicrobial properties of “Napoletano” green and purple basil were analyzed by Tenore et al. [67], who reported that the main phenolic acids in purple basil were rosmarinic, ferulic and gallic acid; while in green basil, the most abundant phenolic constituents were gallic acid, followed by rosmarinic and ferulic acids. Interestingly, the functional molecule rosmarinic acid detected in “Napoletano” type basil was by far higher than what on average has been reported for other common varieties such as “Sweet basil”, “Thai basil”, “Genovese Italiano”, and “Purple Petra” (112, 128, 117, and 352 per 100 g fresh weight, respectively) [59]. The main anthocyanins detected in purple basil extract were cyanidin-based *p*-coumaril and malonil acids, acting as powerful antioxidants with potential use as medicinal agents [68]. Tenore and co-workers also demonstrated in the same study that extracts of “Napoletano” green and purple basil both had a broad antimicrobial spectrum able to reduce the growth of all human pathogenic and food spoilage bacteria and molds tested [67].

Regarding the health-promoting effects of basil, several preclinical and clinical studies showed that extracts from basil, particularly the purple one, may alleviate hyperglycemia associated with type 2 diabetes [69,70]. The anti-diabetic beneficial effects of basil extract may be due in part to catechin and especially to rosmarinic acid, which has been found to inhibit key enzymes such as α-amylase, α-glucosidase, and aldose reductase [71,72,73]. In addition to being anti-diabetic, basil extract may also be an efficient tool against hyperlipidemia by effecting lower cholesterol and triglyceride levels in the blood [74,75]. Reduced uptake of lipids and lower values of total cholesterol and low density lipoprotein may reduce the risk of cardiovascular diseases.

### 2.3. Perilla

Perilla (*Perilla frutescens* L. Briit) belongs to the Lamiaceae family (formely Labiateae) which consists of 235 genera and more than 700 species [76]. Perilla is an edible herb widely consumed in Asian countries such as China, Korea, Japan, and India. Similar to spinach, perilla leaves are also characterized by a high concentration of carotenoids. In fact, according to Müller-Waldeck et al. [77], perilla may contain high contents of carotenoids, especially up to five-fold higher lutein than other carotenoid-rich leafy vegetables. The leaves of perilla contain a range of bioactive phenolic molecules such as caffeic acid, catechin, chrysoeriol, ferrulic acid, luteolin, quercetin, and rosmarinic acid [78,79]. In particular, secondary metabolites such as rosmarinic acid and perillaldehyde (an essential oil constituent) have demonstrated potential to prevent a wide range of diseases particularly owing to their anti-diabetic, anti-depressant, anti-bacterial, anti-cancer, and antimicrobial properties [76]. Thus the concentrations of these two phytochemicals are crucial for their clinical and culinary applications. It is worth noting that these two secondary metabolites are produced in perilla by two different biosynthetic pathways, namely the monoterpene and phenylpropanoid pathways, and may increase independently in relation to the perilla chemotype and abiotic environmental stress conditions [80]. Perilla is present in nature in two main chemical-varietal phenotypes: (i) The red-pigmented cultivar *P. frutescens* var. *crispa*, known as “Zi-So” and widely grown in China where it is used as a spicy herb, leafy vegetable, and medicinal plant, and (ii) the non-pigmented green cultivar *P. frutescens* var. *frutescens* known in Japan as “Shisoyo” or “Shiso” and mainly used as an oil crop but also as ingredient of skin creams and food products [81,82]. According to Meng et al. [78,83], three cinnamic derivates (caffeic acid, coumaroyl tartaric acid, and rosmarinic acid) ranged from 0.1 to 11 mg/g; six flavonoids (apigenin 7-*O*-caffeoylglucoside, apigenin 7-*O*-diglucuronide, luteolin 7-*O*-diglucuronide, luteolin 7-*O*-glucuronide, scutellarein 7-O-diglucuronide, and scutellarein 7-*O*-glucuronide) ranged from 3.5 to 18.5 mg/g; and six anthocyanins (0.7–2 mg/g) including cis-shisonin, cyanidin 3-*O*-(*E*)-caffeoylglucoside-5-*O*-malonylglucoside malonylshisonin and shisonin were detected on eight tested cultivars of perilla. Concerning the health effects of perilla extract, Narisawa et al. [84] reported that perilla leaves carry anti-tumor properties. In their work, the authors showed that treatment of female rats with a 12% fat diet based on perilla extract and safflower oil in 1:3 or 1:1 ratio effected better protection against colon cancers as compared to safflower oil alone [84]. 

Comparing green (Korean cultivar) and red-pigmented (Japanese cultivar) perilla, Rouphael et al. [82] observed that green perilla produced exclusively perilla ketone (PK), whereas the red perilla contained perillaldehyde (PA). Similar results were reported by Martinetti et al. [81] in a study profiling two red-leaf (“Aka Shiso” and “Purple Zi Su”) and three green-leaf cultivars (“Ao Shiso”, “Qing Su”, and “Korean perilla”) with the later containing PK instead of PA. The terpenoid component present in green-pigmented perilla has been demonstrated to be toxic for cattle and horses, since PK is considered a potent lung toxin [77,85], but the health-effect as well as the toxic dose/concentration to humans is still controversial, therefore Müller-Waldeck and co-workers [77] concluded that some Korean genotypes (green-pigmented cultivars) are not suitable/recommended for fresh consumption. Interestingly, several authors showed that PA and PK present in red and green perilla can stimulate the TRPA1 (Transient Receptor Potential) cation channels which are actively involved in multiple biological mechanisms such as pain perception and their functional role in the prevention of certain types of tumor has been proved [86,87].

### 2.4. Swiss Chard

Green chard also known as Swiss chard (*Beta vulgaris* var. *cicla* L.) belongs to the Amaranthaceae-Chenopodiaceae family and is considered an important leafy vegetable grown for its green or reddish leaves and the white, yellow, or red leaf stalk. Green beet belongs to the same family of the root vegetable red beet (*Beta vulgaris* var. *rubra* L.). Traditionally, Swiss chard has been employed for its health-promoting properties as folk remedy for liver/kidney diseases, for triggering the hematopoietic and immune systems and also as a target diet in some tumors treatment [88]. As they grow, Swiss chard leaves accumulate a wide range of macro and micro minerals such as P, K, Ca, Mg, and Fe and several lipophilic vitamins (such as A and E and also carotenoids), as well as hydrophilic vitamins (such as B3, B5, B9, and C) [89]. According to Mzoughi et al. [90] Swiss chard leaves have a nutritional and functional profile catering to modern human diets. In the latter study, Swiss chard leaves were characterized by high concentrations of secondary metabolites such as (myricitrin, *p*-coumaric, and rosmarinic acid), flavonoids, carotenoids (β-carotene, chlorophyll, and lycopene) and some target volatile compounds (decanal, E-anethole, and octanoic acid). Mzoughi and co-workers demonstrated that the high antioxidant capacity on ABTS and DPPH of Swiss chard ethanol extract was accompanied with significant inhibitory effects on α-amylase and α-glucosidase; thus the Swiss chard extract could be explored in the near future as potential functional food with antioxidant and anti-diabetic properties [90].

In a recent review paper, Ninfali et al. [91] reported that green beet extract may regulate the hematic concentration of glucose, decrease lipid peroxidation, lower triglycerides and cholesterol levels, and improve glutathione levels. The health protective secondary metabolites found in *B. vulgaris cicla* have been identified as a class of G-Glycosyl flavonoids including (i) isovitexin, (ii) vitexin, (iii) vitexin-2-*O*-xyloside and iv) vitexin-2-*O*-rhamnoside, which are characterized by high biological activity [91]. According to Lee et al. [92], vitexin is able to reduce drastically the mitochondrial membrane potential in leukemia cell. Similarly, Nifali et al. [93] and Gennari et al. [94] reported that vitexin-2-*O*-xyloside and vitexin-2-*O*-rhamnoside were able to reduce the proliferation rate of MCF-7 breast and RKO cancer cells. Concerning the anti-inflammatory properties of Swiss chard, Borghi et al. [95] demonstrated that the administration of 10 mg/kg of vitexin is able to decrease the levels of pro-inflammatory cytokines. Overall, in vitro and in vivo experiments carried out on animals and humans demonstrated that the biological activity of vitexin, vitexin-2-*O*-xyloside, and vitexin-2-*O*-rhamnoside can trigger the expression of a wide range of genes associated with inhibition of cancer cell proliferation and anti-inflammation activities.

### 2.5. Brassica Leafy Vegetables

The group of brassicaceous leafy vegetables, formerly referred to as cruciferous vegetables, includes a wide range of species with potential health-promoting properties such as kale (*Brassica oleracea* var. *sabellica*), pack choi (*Brassica rapa* var. *chinensis*), mizuna (*Brassica rapa* var. *japonica*), watercress (*Nasturtium officinale* R.Br.), wild and salad rocket (*Diplotaxis tenuifolia* [L.] DC and *Eruca cesicaria* [L.] Cav., respectively). According to FAOSTAT [27] database, roughly 12% of the vegetables grown worldwide are members of the Brassicaceae family. Recent reports [96,97] have suggested that brassicaceous leafy vegetables constitute valuable sources of phytochemicals. They contain high levels of vitamins (C, E [as α- and γ-tocopherols] and K [phylloquinone]), carotenoids, and phenolic compounds. In addition to the latter phytochemicals, *Brassica* leafy vegetables are characterized by sulfur-containing glucosinolates and methylcysteinsulfoxide compounds. The genetic factor is the most important and influential one in terms of modulating the biosynthesis and accumulation of phytochemicals in *Brassica* leafy vegetables [97]. In a comparative study of antioxidant molecules in four *Brassica* leafy vegetables (mizuna, salad rocket, watercress, and wild rocket), the authors observed a large variability in phytochemical concentrations [98]. For instance, watercress showed the highest polyphenol and vitamin C content, while salad and wild rocket were characterized by high concentrations of kaempferol and quercetin derivatives and finally mizuna exhibited significant concentrations of isorhamnetin and sinapic acid [98]. The authors highlighted the potential value of salad *Brassica* leafy vegetables as dietary sources of antioxidants conferring a wide range of positive health effects against type 2 diabetes and cardiovascular diseases. 

Kale is a leafy *Brassica* species considered a potent source of glucosinolates and isothiocyanates, their main breakdown products. A study from the CDC (center of disease control and prevention) reported kale being ranked 15th among “powerhouse” vegetables and fruits [99]. The most abundant glucosinolates in kale were: 3-(methylsulphinyl)propyl, 2-propenyl and also 4-(methylsulphinyl)butyl glucosinolates. Genotypic variation within eight cultivars of kale (“Starbor”, “Beira, “Scarlet”, “Premier”, “Olympic Red”, “Toscano”, “Dwarf Siberian”, and “Red Russian”) revealed that “Beira” and “Olympic Red” were characterized by the highest total concentration of glucosinolates and were proposed as functional foods [100]. In another experiment carried out by Hahn et al. [101] on 25 kale cultivars, the authors observed a great variation in glucosinolate profiles. Similarly, Ferioli et al. [102] reported higher variation in the aliphatic compared to the indole glucosinolates (9- and 5-fold, respectively) across 25 kale cultivars harvested from different European countries (Italy, Portugal, and Turkey). The presence of isothiocyanates in *Brassica* leafy vegetables has been reported to confer anti-diabetic, anti-inflammatory and anti-cancer properties [103,104,105,106,107,108]. Although less popular, even “ornamental cabbage” or kale (*Brassica oleracea* L. var. *acephala* DC.) have genotypes characterized by the accumulation of different pigments [109,110,111]. *Brassica* leafy vegetables, in particular kale, are considered additionally as a rich source of carotenoids (lutein and β-carotene), as well as chlorophylls (a and b). Carotenoid concentrations of 33 kale cultivars were analyzed and quantified [112]. Zeaxanthin was the most abundant carotenoid in 21 cultivars. Moreover, American and hybrid cultivars and accessions were characterized by high concentrations of zeaxanthin, whereas, German landraces, German commercial varieties, Italian, and red-colored kale varieties exhibited high concentrations of chlorophyll a and b [112].

Emerging market trends catering to shifting consumer perceptions of quality [32], have resulted in colored *Brassica* leafy vegetables (e.g., violet kale or pack choi) containing anthocyanins garnering the attention of nutritionists and horticultural scientists. Recently, Mageney et al. [112] proposed that the anthocyanin content could be used as a marker to differentiate between varieties/cultivars. Testing green and red-pigmented pack choi, Zheng et al. [113] observed that red pack choi produced higher concentrations of carotenoids, total phenolic compounds, total flavonoids, glucosinolates, and anthocyanins compared to its green counterpart. Importantly, the regular intake of anthocyanins from such colored leafy vegetables has been positively correlated with the prevention of various liver diseases, and also with the reduction of colon cancer, hepatic inflammation and oxidative stress [114].

The main pigments isolated in the studied leafy vegetables are presented in Table 1.

## 3. Fruit Vegetables

### 3.1. Tomato

Tomato (*Solanum lycopersicum* L.) is an important fruit vegetable, widely consumed all over the world due to its rich nutrient content, special taste, and diverse ways of consumption (fresh, soups, juices, purees, dried, and sauces) [124]. Fruit color and pigments content are two important traits that largely reflect tomato fruit quality, as well as the antioxidant activity which is mainly correlated to the hydrophilic (e.g., soluble phenolic compounds and vitamin C) than to the lipophilic compounds (e.g., carotenoids, vitamin E, and lipophilic phenols) [125]. Fruit color is mainly related to pigments content, such as chlorophyll, carotene, lycopene, phytoene and anthocyanin, and their relative proportions at different maturity stage [126]. The most abundant carotenoid is lycopene, followed by phytoene, phytofluene, ζ-carotene, γ-carotene, ß-carotene, neurosporene, and lutein [127]. Color development is due to the chlorophyll degradation and the synthesis of carotenoids as fruit is developed and ripen. Therefore, the genetic development of tomato fruit color and pigments content is an interesting research area to improve fruit quality and satisfy the diverse consumers’ demands [128].

The consumption of tomato and related food products is associated with the decrease of various diseases incidence such as chronic degenerative diseases, cardiovascular disease, and age-related macular degeneration (AMD) in human health [129]. Raiola et al. [124] reported the nutritional importance of tomato phytochemicals against inflammation processes and prevention of chronic non-communicable diseases (e.g., obesity, diabetes, coronary heart disease, and hypertension). Anthocyanins normally are not produced in tomato fruit, however, some wild tomato species, such as *S. chilense*, *S. cheesmaniae*, *S. lycopersicoides*, and *S. habrochaites* biosynthesize anthocyanins in the sub-epidermal tissue of the fruit, and some alleles from those genotypes have been introgressed into cultivated genotypes [130]. Therefore, combining the dominant Anthocyanin fruit (Aft) gene from *S. chilense* and the recessive atroviolacea (atv) gene from *S. cheesmaniae* into a cultivated tomato background, anthocyanins biosynthesis has been achieved [131,132].

Purple tomatoes have antioxidants and phytochemical properties in both flesh and peel, often in superior levels than those found in conventional red tomatoes [133]. A genetically modified (GM) purple tomato was found to have additional health-promoting effects by prolonging the life of cancer-susceptible mice compared to tomatoes with conventional (red) color [134]. Extracts from fruit of purple tomato (breeding line V118) showed significant and dose dependent anti-inflammatory effect against paw edema in an in vivo study with rat models (edema inhibition: 7.48%–13.8%), suggesting that anthocyanins may play a role in the anti-inflammatory effect [135]. Interestingly, during the last 20 years there has been an increasing interest in developing highly consumed food, such as flavonoids-rich tomato fruit. To that direction, transgenic approaches have been applied to modify the biosynthesis of phenylpropanoids, in order to alter the tomato flavonoid biosynthesis [130].

Phenolic content is varied at different developmental stages of tomatoes, as sun black (SB) tomato had 5.8 and 8.6 mg GAE/g dw phenolic content at mature green and red ripening stage, respectively, contents that were 152% and 134% higher than wild type (WT) [130]. Li et al. [136] reported similar total phenolic content (659.11 mg GAE/100 g dw) for purple tomato as has been reported for other tomato varieties (from 290 to 500 mg GAE/100 g dw) [137]. Individual components of phenolics may also vary among purple and red varieties, as the main phenolic compounds content (chlorogenic acid, naringenin, and rutin) was higher (65.56, 12.82, and 52.39 mg/100 g dw, respectively) [136] in purple tomatoes compared with red tomatoes were chlorogenic acid, naringenin, and rutin content was 2.67, 1.84, and 6.61 mg/100 g dw [138] and 16.7, 2.2, and 16.9 mg/100 g dw, respectively [139]. Apart from chlorogenic acid, naringenin, and rutin Li et al. [136] reported other phenolic compounds in purple tomatoes such as p-coumaric acid (15.68±0.74 mg/100 g dw), gentistic acid (15.25 ± 0.76 mg/100 g dw), ferulic acid (14.51 ± 0.99 mg/100 g dw), caffeic acid (13.65 ± 0.83 mg/100 g dw), and protocatechuic acid (8.95 ± 0.16 mg/100 g dw). Indeed, chlorogenic acid content depends on the developmental stage of fruit and varied from 0.5 to 1.3 mg/g dw in sun black (SB) tomato extracts [130]. Moreover, composition of flesh and peel in tomato mutants differed as flesh contained chlorogenic acid (7.96 ± 0.75 mg/100 g fw), quercetin (5.03 ± 1.02 mg/100 g fw), luteolin (0.45 ± 0.01 mg/100 g fw), and total phenolics (1.18 ± 0.06 mg/g fw), while in peel the respective compounds content was: chlorogenic acid (8.43 ± 0.15 mg/100 g fw), quercetin (5276.15 ± 15.10 mg/100 g fw), luteolin (21.28 ± 1.07 mg/100 g fw), and total phenolics (5.95 ± 0.27 mg/g fw) [133]. 

Among the several phytochemicals identified in plants, *cis*- and *trans*-resveratrol (3,4,5-trihydroxystilbene) are polyphenols that belong to the stilbene class; however, only the *trans* form is biologically active in the human body [140]. Numerous biocidal activities exerted by resveratrol have been reported such as antioxidant, antidiabetic and estrogenic activity, anticancer effects through the preservation of the regular cell cycle, the inhibition of tumor invasion and angiogenesis, and cardiovascular effects through the reduction of the expression of endothelial adhesion cells and the inhibition of cell apoptosis and platelet aggregation [141,142,143,144,145]. However, resveratrol’s daily intake by humans has still to be established [143]. Vagula et al. [146] quantified *trans*-resveratrol in *S. americanum* Mill. fruit, which ranged between 1.07 and 0.796 μg/g for fruit pulp and peel, respectively, and these levels were significantly higher when compared to freeze-stored fruit (0.1353 μg of *trans*-resveratrol/g of sample) and to other berries [146].

Vasco et al. [147] reported the higher antioxidant capacity of purple (purple-red variety) tamarillo or tree tomato (*Solanum betaceum* Cav.), compared to the golden-yellow variety and reported 9.3 μmol trolox/g fw, 3.0 μmol trolox/g fw and 40 μmol trolox/g fw for seed-jelly, pulp, and peel tissues of purple fruits compared to 3.8 μmol trolox/g fw, 2.3 μmol trolox/g fw and 22 μmol trolox/g fw for seed-jelly, pulp and peel tissues of yellow fruits, respectively. Similarly, Sestari et al. [133] reported increased antioxidant capacity (DPPH) in peel (38.12 ± 4.27 μmol trolox/g fw) compared to flesh (8.64 ± 0.45 μmol trolox/g fw) in tomato mutants. Interestingly, the oxygen radical absorption capacity assay (ORAC) value for the hydrophilic extracts in purple tomato reported by Li et al. [136] was 323.23 μmol trolox/g dw, which was 2-fold higher than the ORAC value of the traditional tomato cultivar San Marzano (140 μmol trolox/g dw) reported by Ninfali et al. [148]. Similar observations were made by Blando et al. [130] who stated 3-fold higher trolox equivalent antioxidant capacity (TEAC) value (31.6 μmol trolox/g dw) in sun black tomatoes compared with the wild type (10.3 μmol trolox/g dw) at the red-ripening stage. Moreover, antioxidant capacity of purple fruit was higher at the ripe (red ripe stage-RR) fruit compared to the unripe (mature green stage-MG) ones, probably explained by the great increase in polyphenols accumulation during ripening (from 5.8 to 8.6 mg GAE/g dw, in MG and RR, respectively) [130]. In the same study, total ascorbic acid content was higher in sun black than wild type fruits (37.3 ± 1.4 vs. 27.1 ± 1.1 mg 100/g fw, respectively) [130].

The antioxidant capacity of fruit is not only related to phenolics and ascorbic acid but also to the carotenoids, flavonoids, and anthocyanins content. Lycopene, the main phytochemical of tomatoes, is known for its important role in human health related functionalities [149]. Lycopene supplementation in an in vivo study with iodoacetamide-induced colitis rats showed reduced tissue malondialdehyde (MDA) levels, the histological signs of colon injury, and increased superoxide dismutase levels in the red blood cells [150]. In the study Li et al. [136], lycopene was the dominant carotenoid (185.01 μg/g dw) in breeding line V118, followed by β-carotene (47.11 μg/g dw) and lutein (2.66 μg/g dw). About 8.1% of the total carotenoids in V118 were *cis*-carotenoids, a lower value compared to that of most of the tomato varieties studied [136]. Moreover, Li et al. [128] reported that purple fruit (cv “Zi Ying”) had increased antioxidant capacity compared to green fruit (cv “Lv Ying”), with lycopene content of 36.51 ± 2.86 mg/kg fw and β-carotene 13.38 ± 1.31 mg/kg fw in purple fruit versus lycopene content of 1.35 ± 0.05 mg/kg fw and β-carotene 6.80 ± 0.32 mg/kg fw in green fruit. In a comparative study, Hazra et al. [151] pointed out the dietary role of purple tomato (AftAft dgdg genotype) due to the increased values in ascorbic acid (31.56 ± 2.41 mg/100 g fw), lycopene (6.13 ± 0.39 mg/100 g fw), β-carotene (0.65 ± 0.14 mg/100 g fw), and anthocyanin (20.73 ± 2.86 mg/100 g fw), compared to the overall mean value of 31 hybrids. 

Li et al. [136] reported a total carotenoid content of breeding line V118 of 234.78 μg/g dw, being within the range of the average amounts reported for red tomatoes (132–583 μg/g dw) [152]. At the red ripe stage, total carotenoids did not differ between the sun black (SB) and wild type (WT) tomatoes; however, the β-carotene content was significant higher in the SB sample, whereas the lycopene content was lower [130]. Similarly, Vasco et al. [147] reported higher β-carotene levels in purple tamarillo than in yellow tomato variety. Generated double and triple mutants (Anthocyanin fruit/high pigment 2(Aft/hp2) and Anthocyanin fruit/atroviolacium/high pigment 2 (Aft/atv/hp2)) of purple tomatoes had higher lycopene and β-carotene levels and up to 63% of vitamin C compared to tomato cultivar Micro-Tom, suggesting accumulating trends of relevant phytochemicals in near-isogenic lines [133]. 

Anthocyanins, the most abundant flavonoid constituents in pigmented fruit and vegetables, posse potential health beneficial effects, such as antioxidant, anti-inflammatory, anticancer, and antidiabetic activities [153,154]. Anthocyanins also had notable effects against inflammation by inhibiting cyclooxygenase-2 (COX-2) expression, inducible nitric oxide protein and mRNA expression [155]. The “Giant” and “New Zealand” purple cultivars, had total anthocyanins content of 102.35 ± 1.46 mg/100 g dw and 168.88 ± 2.65 mg/100 g dw, but also revealed high antioxidant activity which might be related to their overall phenolic composition [156]. Zhang et al. [157] reported the role of anthocyanins in postharvest storage of tomatoes as in purple tomatoes, anthocyanins doubled the self-life of fruit by delaying over-ripening and reducing susceptibility to *Botrytis cinerea*.

Li et al. [136] through an LC-MS study reported three major anthocyanins, which were mainly acylglycosides of petunidin and malvidin. Among these anthocyanidins, petunidin was the predominant aglycone (91.9%), and the rest of the minor aglycones accounted for only 9.1% of the total anthocyanidins [136]. Moreover, petunidin is not usually synthesized in vegetables and fruit, and little is known about its health benefits, however in tomato mutants petunidin revealed considerable amounts (>60 mg/100 mg fw) in fruit peels of the lines combining Aft and hp2 genes [133]. The total anthocyanin content in breeding line V118 was 72.31 mg/100 g dw, including 9.04, 50.18, and 13.09 mg/100 g dw of petunidin-3-*O*-caffeoyl-rutinoside-5-*O*-glucoside, petunidin-3-*O*-(*p*-coumaryl)-rutinoside-5-*O*-glucoside, and malvidin-3-*O*-(*p*-coumaryl)-rutinoside-5-*O*-glucoside, respectively [136]. Moreover, Blando et al. [130] reported that petanin (Petunidin 3-(6-(4-(*E*-*p*-coumaroyl)rhamnosyl)glucoside)-5-glucoside (petanin)) and negretein (Malvidin 3-(6-(4-(*E*-*p*-coumaroyl)rhamnosyl)glucoside)-5-glucoside) represented 56.6% and 21.4% of the total anthocyanins content in sun black (SB) fruit peel, respectively, whereas no anthocyanins were detected in wild type (WT) tomato fruit. 

The content of anthocyanins in the Del/Ros1 transgenic tomato is equally distributed within fruit, with 5.1 ± 0.5 g/kg dw being detected in the peel and 5.8 ± 0.3 g/kg dw in the flesh, but not detected in seeds [7]. These values are higher than those reported for well-known anthocyanin-rich foods such as red raspberry (3.9 g/kg dw; [158]), strawberry (3.2 g/kg dw; [158]), and mulberry (2.1 g/kg dw; [159]). In a study with transgenic plants, the predominant anthocyanins in the Del/Ros1 transgenic tomato were delphinidin-3-(trans-coumaroyl)-rutinoside-5-glucoside and petunidin-3-(trans-coumaroyl)-rutino side-5-glucoside, which contributed to nearly 86% of the total anthocyanins content, while two new anthocyanins, malvidin-3-(p-coumaroyl)-rutinoside-5-glucoside and malvidin-3-(feruloyl)-rutinoside-5-glucoside making up to 6% of the total anthocyanins content, were also reported [7]. Three mutant genes have been identified that can lead to the production of anthocyanins in the peel of the fruit, namely Anthocyanin fruit (Aft), Aubergine (abg), and atroviolacea (atv), while the Aft gene was also identified in crosses with *Solanum chilense* Dunal [160]. This gene is located in chromosome 10 and its presence in tomato leads to the production of anthocyanin pigments, mainly delphinidin, malvidin and petunidin, as well as to higher levels of the flavonols quercetin (3.6-fold), and kaempferol (2.7-fold), in tomato fruit [160,161].

Tamarillo crop is attracting research interest lately due to the high content in antioxidants and phytochemicals. The tamarillo, a non-climacteric edible fruit, is quite popular in local markets, especially in South America, consumed in juices or fresh and being highly appreciated due to high polyphenols levels [156], β-carotene (provitamin A), vitamin B6, vitamin C (ascorbic acid), vitamin E, and iron contents [162]. Among phenols, the presence of anthocyanins (delphinidin, cyanidin, and pelargonidin glycosides) and hydroxycinnamoyl derivatives (e.g., 3-*O*-caffeoylquinic acids, caffeoyl glucose and feruloyl glucose) have been described in several reports [147,163,164], while recently rosmarinic acid has been also identified [156]. The hydroxycinnamoyl derivatives show antioxidant properties and have been related to protective effects on human health [165]. In particular, the caffeoyl ester of rosmarinic acid has various biocidal activities, such as antiviral, antibacterial, anti-inflammatory, and antioxidant effects [166]. Other compounds were tentatively identified as different rosmarinic acid glucosides, caffeoyl glucoside, feruloyl glucoside, and ferulic acid dehydrodimers. Pelargonidin 3-*O*-rutinoside and delphinidin 3-*O*-rutinoside were the main anthocyanins in purple cultivars of tomato fruit [167]. Vasco et al. [147] reported anthocyanins content of 38 mg/100 g fw in purple tamarillo which was higher than previous reports in yellow fruit (8.5 mg/100 g fw) [167].

Both *Solanum americanum* Mill. and *S. villosum* Mill. are important medicinal plants of the Solanaceae family, however the blackish-purple (*S. americanum*) and reddish-orange (*S. villosum*) colored fruit are mostly consumed in India, Ethiopia, Ghana, China, and Brazil [168]. Mohy-Ud-Din et al. [169] reported the different important steroidal glycoalkaloids like β-Solamargine, α-Solamargine, Solasonine, α-Solanine, solasodine, and Solanidine, with latter being well-recognized for its anticancer activities [170].

### 3.2. Eggplant

Eggplant (*Solanum melongena* L.) fruit are very popular vegetables grown worldwide in subtropical and tropical regions [171]. They considered as one of the top 10 vegetable in terms of antioxidant capacity [172] and contain a variety of antioxidants and phytochemicals such as, ascorbic acid, phenolics, and flavonoids that provide health benefits [173]. The most abundant phenolic compound is 5-*O*-caffeoylquinic acid, known as chlorogenic acid (ChA), which is considered as the main contributor to the overall antioxidant capacity [174,175]. However, eggplant fruit are poor sources of provitamin A and vitamin E, with average values of 27 IU 100/g fw and 0.30 mg/100 g fw, respectively [173]. American purple fruit are the most commonly marketed type, though white cultivars have gained consumers acceptance in recent years.

Eggplants are quite versatile vegetables and could be subjected to a number of different processing and cooking methods which may further affect fruit antioxidant capacity [176]. Akanitapichat et al. [173] reported that the antioxidant activities of eggplant were correlated (*r* = 0.531–0.796) with the total amounts of phenolics and flavonoids. Significant correlation was found between hepatoprotective activities and total phenolics/flavonoids content (*r* = 0.637–0.884) and antioxidant activities (*r* = 0.585–0.958), indicating the contribution of the polyphenols present in eggplant to its hepatoprotective effect (human hepatoma cell line HepG2) against tert-Butylhydroperoxide (t-BuOOH)-induced toxicity [173]. Akanitapichat et al. [173] reported that total phenolics content in purple fruit was of 1002.67 ± 8.33 mg GAE/100 g extract. Nisha et al. [176] suggested a higher content of total phenolics and anthocyanins in a purple small-sized fruit variety (106.98 mg/100 g fw and 0.756 mg/100 g fw, respectively) than the three other examined varieties (purple moderate-sized fruit (80.31 mg/100 g fw and 0.525 mg 100/g fw, for total phenolics and anthocyanins, respectively), green long-sized (50.79 mg/100 g fw and 0.0475 mg/100 g fw, for total phenolics and anthocyanins, respectively), and purple big-sized (49.02 mg/100 g fw and 0.53 mg/100 g fw, for total phenolics and anthocyanins, respectively)). In the same study, it was reported that purple small-sized variety revealed the greatest antioxidant activity. 

Flavonoids represent only about 10–15% of total phenolics [177] and hydroxycinnamic acid derivatives, particularly free chlorogenic acid is the major phenolic antioxidant regardless of the genotype [177,178]. However, the chlorogenic acid content in eggplant is influenced by both genetic and environmental factors, including the fruit developmental stage, cultivar, and crop and postharvest management [179]. For example, the chlorogenic acid content was 16% higher in purple eggplants compared to white eggplant slices [180]. According to Akanitapichat et al. [173] who compared five eggplant varieties, the purple fruit had higher total flavonoids content (3954.2 ± 6.06 mg catechin equivalents-CE/100 g extract) content compared with green and white ones. Sadilova et al. [181] reported that flavonoids isolated from *S. melongena* showed potent antioxidant activity against chromosomal aberrations induced by Doxorubicin. The purple eggplant had high antioxidant activity of DPPH and ABTS with EC_50_ of 66.74 ± 4.60 μg/mL and 53.18 ± 0.71 μg/mL, compared with green and white varieties [173]. 

In purple-fruited genotypes of pepper and eggplant the abundance of anthocyanin levels is superior in unripe fruits and decrease upon ripening, often to complete disappearance [182]. It is noteworthy that eggplant fruit reaches its commercial maturity long before its physiological ripeness, as practically it is harvested at the immature fruit stage [183]. The anthocyanin concentration in the purple fruit eggplant cultivars is higher in comparison to other deeply colored fruits and vegetables, e.g., 2.34-fold that of grapes, and 7.08-fold that of red onions [5]. In purple pigmented eggplants, the antioxidant anthocyanins (delphinidin derivatives) is limited as found at the peel tissue which represents less than 5% of the total fruit weight [178,184]. Examining the anthocyanins content of wild type (WT), purple-black (S9-1), green (L6-4), and white (U36-1) eggplants, Xi-Ou et al. [185] found that the anthocyanin content of purple-black (S9-1) was higher than that in WT eggplant, while green eggplant (L6-4) had the lowest levels of anthocyanins. In another study, Zhang et al. [186] reported the total anthocyanins content from purple eggplant (cv Zi Chang) skin to be 1.24 mg/g dw. Moreover, nasunin (delphinidin-3-(*p*-coumaroylrutinoside)-5-glucoside), an anthocyanin isolated from the skin of purple eggplant fruit, is associated with both inhibition of hydroxyl radical generation and superoxide scavenging activity [173,187].

Extracts from eggplant fruit skin were demonstrated to possess high capacity in scavenging of superoxide free radicals and inhibiting hydroxyl radical generation by chelating ferrous iron [187]. Additionally, eggplant extract resulted in hypolipidemic activity in rats fed normal as well as high fat diets [188], suppressed tumor growth and metastasis [189], and inhibited inflammation that can lead to atherosclerosis [190]. Not only fruit but various parts of the plant are useful in the treatment of inflammatory conditions, cardiac debility, neuralgia, ulcers of nose, cholera, bronchitis, and asthma, while they possess analgesic and hypolipidemic properties [191]. *S. melongena* is also a natural source of vitamin A affecting the eye health in children [192].

### 3.3. Pepper

Pepper (*Capsicum* spp.) is one of the oldest domesticated and utilized crops and the genus *Capsicum* consists of approximately 31 species of which the five domesticated species are *C. annuum*, *C. baccatum*, *C. chinense*, *C. frutescens*, and *C. pubescen* [193]. Average world production and cultivated area of dry and green peppers are estimated at 3.9 and 34.5 million tons respectively, harvested from 1.8 and 1.9 million hectares respectively [27]. Pepper fruit have high nutritive value, as they are rich in vitamin C (ascorbic acid), provitamin A (β-carotene), vitamin E (tocopherols), flavonoids and capsaicinoids, and other carotenoid pigments such as lycopene and zeaxanthin [194]. The noticeable level of phenolic compounds and carotenoid pigments also contributes to the antioxidant properties of sweet pepper [195].

Immature fruits are usually colored in white, green, purple, and black shades and gradually color changes to yellow, orange, red and brown as fruit maturity advances [196,197]. The differences in fruit color is mainly due to the differential accumulation of flavonoids and carotenoids [198]. Anthocyanin accumulation in the outer epidermis of immature pepper fruit is responsible for the purple or black color at 30 d after anthesis [199] and turns to red color at 50 d after anthesis [182].

Liu et al. [182] compared the flavonoids biosynthesis at 30 d after anthesis for green, white, and purple varieties, and reported that anthocyanins, flavones, and flavonols content was significantly higher in purple variety than in the other varieties, with delphinidin, luteolin, chrysoeriol and quercetin derivatives being the most abundant polyphenols. Delphinidin, cyanidin, and malvidin derivatives were the major anthocyanins in colored peppers among the 16 anthocyanins detected of which delphinidin 3,5-diglucoside and delphinidin 3-*O*-rutinoside were specifically accumulated in purple peppers [182]. In the same study, it was reported that the purple color of fruit is related to the high accumulation of cyanidin and delphinidin derivatives at 30 d after anthesis.

In chili peppers, anthocyanins’ presence and pigmentation of purple or black and magenta is also possible, and is usually found in flowers, fruit, and foliage [200]. Several reports highlighted the presence of anthocyanins in chili pepper fruit, but so far delphinidin is the only anthocyanidin identified [200,201]. Similarly, Sadilova et al. [181] reported delphinidin-3-*trans*-coumaroylrutinoside-5-glucoside (nasunin 89%) and delphinidin-3-*cis* coumaroylrutinoside-5-glucoside (4.6%) as the main anthocyanins (averaged at 320 μg/g fw) in German chili pepper (*C. annuum* L.), while similar results were found in two Mexican chili peppers [202]. Moreover, hydroponically grown dark violet pepper (cv. “Zorro”) had the highest concentration of quercetin and catechin when compared to orange, red and yellow fruit cultivars [203].

Capsanthin-capsorubin synthase (CCS), as a unique enzyme in pepper and tiger lily, converts antheraxanthin and violaxanthin into capsanthin and capsorubin, respectively [204]. Although the exact mechanism is under investigation, Liu et al. [182] reported that the highly active CCS drives antheraxanthin to be converted into capsanthin in purple fruit, which reduces the flux to violaxanthin, eventually resulting in significantly lower levels of antheraxanthin and violaxanthin in purple than in green and white fruit varieties.

### 3.4. Lablab and Common Bean

Lablab (*Lablab purpureus* L.), an ancient legume species, serves as a vegetable and is widely cultivated throughout the tropics, subtropics and temperate zones [205]. Fruit are green pods, 6 cm long by 2 cm wide, flattened, contain 4–5 seeds and turn light brown-purple when mature [206]. Al-Snafi [207] and Momim et al. [208] reviewed the phytochemical properties of lablab and its medicinal importance, exhibiting antidiabetic, anti-inflammatory, analgesic, antioxidant, cytotoxic, hypolipidemic, antimicrobial, insecticidal, hepatoprotective, antilithiatic, antispasmodic effects. Moreover, Momim et al. [208] and Deoda et al. [209] reported that the juice derived from the fruit pods was used as astringent, digestive, stomachic, to expel worms and for the treatment of inflamed ears and throats. Soetan [210] studied the pharmacological potentials of three varieties (“Rongai brown”, “Rongai white”, and “Highworth black”) of *L. purpureus* seeds and showed that raw and aqueous extracts contained various phytochemicals including trypsin inhibitors, hemagglutinin, cyanogenic glycosides, oxalates, phytates, tannins, and saponins, with greater contents in raw material compered to aqueous extracts. Other biocidal effects have been reported including antilithianic activity [209], hepatopreotective effects [211], and inhibited trypsin and plasmin activity [212].

Momim et al. [208] reported significant antioxidant capacity (DPPH) with the lowest IC_50_ found in purple lablab compared to the white one (430.00 μg/mL *vs* 853.13 μg/mL). In the same study, total flavonoids content in purple fruit was 32.09 ± 0.36 mg quercetin equivalent/g fw while in green lablab it was 42.55 ± 5.77 mg quercetin equivalent/g fw. Bhaisare et al. [206] reported vitamin C content of 81.00 ± 0.16 mg/g and vitamin E content of 73.66 ± 0.08 mg/g in fresh bean seeds of *L. purpureus*. 

Total content of anthocyanins in purple (cv. “Hong Fu”) pods was about 1.58 mg/g, while low amounts were detected in green (cv. “Qing Feng”) ones. Compared to green pods, five kinds of anthocyanins (malvidin, delphinidin, and petunidin derivatives) were found in purple pods by HPLC-ESI-MS/MS and the major compounds were identified as delphinidin derivatives [213]. Besides, nine kinds of polyphenol derivatives, namely quercetin, myricetin, kaempferol, and apigenin derivatives were detected by UPLC-ESI-MS/MS and the major components were quercetin and myricetin derivatives [213].

Common bean (*Phaseolus vulgaris* L.) is another legumes species with pods of varied colors, including black, red, blue, and violet [214,215]. Anthocyanins content may vary significantly depending on the genotype, while polyphenols content is highly associated with the antioxidant activities of pods [216]. Tsuda et al. [217] reported that pelargonidin-3-glucoside, cyanidin-3-glucoside, and delphinidin-3-glucoside isolated from *P. vulgaris* (black bean) seed coat, as well as their standard aglycones, have strong antioxidative activity in a liposomal system and reduced formation of malondialdehyde by UVB irradiation [217]. According to Mazewski et al. [218], purple beans contain mostly condensed tannins which are responsible for the antiproliferative activities against human colon cancer cell lines (HCT-116 and HT-29).

### 3.5. Pepino

Pepino (*Solanum muricatum* Aiton), a close relative to tomato and potato, is an herbaceous Andean domesticated species grown for its juicy, sweet, and aromatic fruit, with increasing commercial and export interest in South America from exotic fruit markets [219]. Fruit color may be white, cream, yellow, maroon, or purplish [220]. Unripe pepino fruit is green while 51 days from fruit set, newly-acquired purple stripes appear, resulting in fruit softening along with decreases in total pectin and hemicellulose content [221]. 

Various health benefits were revealed for pepino, including treatment of diabetes, stroke, high blood pressure, heartburn (indigestion), cancer, kidney, constipation, and hemorrhoids [220], activities mostly attributed to the significant amounts of vitamin C, carotenoids, and phenolics [222]. Moreover, Hsu et al. [222] reported the antioxidative, anti-inflammatory, and antiglycative effects of pepino extract. In the same study, aqueous and ethanol extracts had similar content of total phenolic acids (averaged at 1145 mg/100 g dw) but aqueous extracts were richer than ethanol extracts in terms of ascorbic acid (43.8 vs. 6.6 mg/100 g dw), total flavonoids (875 vs. 461 mg/100 g dw), cinnamic acid (75.7 vs. 23.0 mg/100 g dw), ferulic acid (82.3 vs. 11.8 mg/100 g dw), rosmarinic acid (47.2 vs. 8.4 mg/100 g dw), quercetin (126.5 vs. 90.3 mg/100 g dw), and naringenin (57.2 vs. 14.7 mg/100 g dw) [222]. 

The main pigments isolated in the fruit vegetables are presented in Table 2.

## 4. Other Vegetables

### 4.1. Broccoli and Cauliflower

Broccoli (*Brassica oleracea* L., var. *italica* Plenk) and cauliflower (*B. oleracea* L., var. *botrytis*) are the two most popular vegetable crops belonging to the Brassicaceae family. Native of the Mediterranean Basin, both species are adapted to a wide range of environmental conditions and are cultivated in all five continents, with an annual production that reached about 26 million tons in 2017, from an estimated harvested area of over 1.39 million hectares worldwide [27]. Primarily known and appreciated for their typical organosulfur compounds [224,225,226], broccoli, cauliflower, and other *Brassica* species are also a rich source of anthocyanins which are responsible of the purple pigmentation of some varieties [18,227]. Typically green, some cultivars and populations of broccoli are characterized by a purple pigmentation of the sepals of the inflorescence [227,228]. Branca et al. [228] found high levels of anthocyanins in a Sicilian broccoli landrace called “Broccolo nero” (Black broccoli) grown around Mount Etna and characterized by a dark violet pigmentation of the inflorescence, stem, and leaf midribs. In a recent work, Yu et al. [227] identified and mapped a major locus and two minor loci associated with the purple sepal trait in broccoli, while the authors hypothesized that the development of purple color may be induced by cold temperatures. In another recent study, Rahim et al. [229] working on the hypocotyl of young green and purple broccoli seedlings identified seven putative candidate genes (BoPAL, BoDFR, BoMYB114, BoTT8, BoMYC1.1, BoMYC1.2, and BoTTG1) responsible for the biosynthesis of anthocyanins. Among those, BoTT8 was expressed considerably more in purple hypocotyl compared to the green ones. Testing the in vitro cytotoxic effect of Sicilian black broccoli stem and leaf extracts at different concentrations (0.05%, 0.1%, 0.5%, 1%, and 5%) against HT29 (colon cancer) and A2058 (melanoma cancer) cells after 24 h treatment in presence or not of the myrosinase enzyme (responsible for the hydrolyzation of glucosinolates), Terzo et al. [230] found that the juice was less toxic in presence of myrosinase especially at higher concentration of the extract (1%–5%), suggesting that factors other than the glucosinolate content, such as polyphenols (including anthocyanins) could be responsible for the cytotoxic effects against HT29 and A2058 cancer cells. Examining the anthocyanin profile of three cultivars of heat-tolerant purple sprouting broccoli, Rodríguez-Hernández et al. [231] found that cyanidin 3-*O*-diglucoside-5-*O*-glucoside derivatives were the major acylated anthocyanins, and each cultivar and plant portion (leaves, inflorescence) had a particular prominent acylated anthocyanin. The same study revealed that compared to green broccoli cv. “Marathon”, purple sprouting broccoli was characterized also by higher levels of glucosinolates. Similarly, Verkerk et al. [232] observed exceptionally high glucoiberin content (396.5 µmol/100 g FW) in purple sprouting broccoli cv “Bordeaux” compared to other green broccoli genotypes, which suggests that there is some sort of interaction between purple pigmentation and glucosinolate profile of broccoli. In another study, analyzing the acylated anthocyanin profile of purple sprouting broccoli and three other green broccoli varieties at the sprouting stage, Moreno et al. [233] observed a significantly higher content of anthocyanins in the purple genotype compared to the green ones and observed that the quantity and quality of anthocyanin pigments were highly variable among the tested genotypes. Out of seventeen anthocyanins identified in the four genotypes only three isomers were predominant in all the genotypes examined: Cyanidin 3-*O*-(acyl)diglucoside-5-*O*-glucoside, cyanidin 3-*O*-(acyl1)(acyl2)diglucoside-5-*O*-glucoside, and cyanidin 3-*O*-(acyl1)(acyl2)diglucoside-5-*O*-(malonyl)glucoside. The purple sprouting genotype was characterized by a higher content of cyanidin 3-*O*-(sinapoyl)(sinapoyl)diglucoside-5-*O*-glucoside, cyanidin 3-*O*-(sinapoyl)diglucoside-5-*O*-glucoside, cyanidin 3-*O*-(feruloyl)diglucoside-5-*O*-glucoside, cyanidin 3-*O*-(sinapoyl)(feruloyl)diglucoside-5-*O*-(malonyl)glucoside, and cyanidin 3-*O*-(sinapoyl)(sinapoyl)diglucoside-5-*O*-(malonyl)glucoside (Table 3). In agreement with previous studies, Moreno et al. [233] concluded that broccoli sprouts could be an excellent source of bioactive compounds rich of flavonoids, including acylated anthocyanins, along with glucosinolates, vitamins, and minerals [234,235,236], and that future studies should evaluate the potential of further enhancing the content of bioactive compounds in broccoli sprouts. 

In the case of cauliflower, while most of the cultivars have been traditionally selected for their white curds [237], many local landraces and commercial cultivars are characterized by colored heads with characteristic pigmentation ranging from green to dark violet. In Italy, green cauliflowers are traditionally grown in Lazio and Marche, while dark violet selections are typically grown in Sicily, Puglia, and other Southern regions characterized by high levels of solar radiation which make it more challenging to produce white curds as traditionally required by the European market [228,238]. Lately, the interest for colored cauliflower varieties substantially increased due to the potential health-beneficial properties of the phenolic compounds that provide the pigmentation of plant tissues [239]. Anthocyanins are in fact responsible for the purple–violet pigmentation also in the case of cauliflower and are considered highly beneficial for human health [8]. The biosynthesis of anthocyanins in cauliflowers is regulated mainly at transcriptional level, and in a particular purple cauliflower mutant it has been demonstrated that the tissue-specific activation of the gene BoMYB2 up-regulated the expression of both BobHLH1 and BobHLH2, leading to the formation of a complex regulation network MYB–bHLH–WD40 (MBW), consisting of MYB, basic Helix-Loop-Helix (bHLH), and WD40 proteins, which in turn activates the structural genes responsible for the biosynthesis of anthocyanins [18,240]. β-carotene accumulation has been also observed in cauliflower curds due to a rare carotenoid gene (Or orange) mutation that activates the biosynthesis of carotenoids in tissues that otherwise would be white [241,242]. Nevertheless, such mutation received limited attention at commercial level and is more relevant to advance our understanding of the carotenoid biosynthesis regulation [243,244].

Analyzing by LC–MS/MS nine Sicilian landraces of violet cauliflower, Scalzo et al. [19] identified cyanidin-3-(6-p-coumaryl)-sophoroside-5-glucoside as the main anthocyanin along with p-coumaryl and feruloyl esterified forms of cyanidin-3-sophoroside-5-glucoside. Scalzo et al. [19] and Kapusta et al. [245] examined also the stability of anthocyanins after processing (blanching, microwave-heating, convection steaming and freezing, and conventional water cooking) and observed substantial changes with the formation of isomers from cyanidin-3-sophoroside-5-glucoside rather than the hydrolysis of anthocyanins, suggesting good stability especially after microwave-heating which could be interesting for food processing applications.

### 4.2. Cabbage and Kale

Among the *Brassica* species, cabbage (*Brassica oleracea* L. var. *capitata*) and Savoy cabbage (*B. oleracea* L. var. *sabauda* L.) are other two popular cole crops grown all over the world for their “heavy” heads constituted by leaves surrounding the terminal buds and that can be green or red-purple [246]. As for broccoli and cauliflower, the pigmentation of red cabbage genotypes is due to the accumulation of anthocyanins. Comparing four green and four red cabbage genotypes, Yuan et al. [247] observed that the structural genes involved in the biosynthesis of anthocyanins (CHS, F3H, F30H, DFR, LDOX, and GST), were steadily up-regulated in red genotypes for the entire growing period. The same authors observed that the expression of the structural genes responsible for the biosynthesis of anthocyanins was up-regulated in correspondence of nitrogen and phosphorous deficiency. Consistently with the mechanism of transcriptional regulation observed in purple cauliflower, in correspondence of the structural gene up-regulation it was observed a simultaneous increase of the transcript levels of the bHLH gene BoTT8, and of the MYB transcription factor BoMYB2. In a recent study analyzing the gene associated with the purple pigmentation of ornamental cabbage characterized by green external leaves and inner purple leaves, Jin et al. [109] found that phytoregulators such as abscisic acid (ABA) and ethylene (ET) play a key role in promoting the biosynthesis of anthocyanins. The same study identified 14 and 19 putative candidate genes involved in the biosynthesis of ABA and ET, respectively, and among those two ABA-biosynthesis related genes (BoNCED2.1, BoNCED2.2) and two ET-biosynthesis related genes (BoACS11, BoACO4) were expressed significantly more in purple leaves than in green leaves and were strongly correlated with the total anthocyanin content of the purple inner leaves.

Analyzing the anthocyanin profile of red cabbage using HPLC/DAD-ESI/Qtrap MS, Arapitsas et al. [248] separated and identified up to 24 anthocyanins all characterized by cyanidin as aglycon, mono- and/or di-glycoside, non-acylated, or acylated with aromatic and aliphatic acids. Similarly, using HPLC-DAD-MS/MS, Wiczkowski et al. [249] identified twenty cyanidin derivatives, with cyanidin-3-diglucoside-5-glucoside as the base structure, and cyanidin-3-diglucoside-5-glucoside, cyanidin-3-(sinapoyl)(sinapoyl)-diglucoside-5-glucoside, and cyanidin-3-(*p*-coumaroyl)-diglucoside-5-glucoside were the most abundant non-acylated anthocyanins. Moreover, Koss-Mikołajczyk et al. [250] identified nineteen different cyanidin derivatives, with cyanidin-3-(feruloyl)-diglucoside-5-glucoside and cyanidin-3-(sinapoyl)(sinapoyl)-diglucoside-5-glucoside having been the most predominant. Similar results were obtained by other authors, who however using different analytical procedures and equipment identified a lower number of anthocyanins [19,251].

Analyzing raw and pickled red cabbage, consistently with other studies McDougall et al. [252] identified eighteen anthocyanin structures, most of which had cyanidin-3-diglucoside-5-glucoside as the core structure non-acylated, mono-acylated or di-acylated with *p*-coumaric, caffeic, ferulic and sinapic acids, but pelargonidin-3-glucoside and new cyanidin-3-*O*-triglucoside-5-*O*-glucoside di-acylated with hydroxycinnamic acids were also identified. The same authors examining the stability of anthocyanins after simulated gastrointestinal digestion found that anthocyanin structures were quite stable, and acylated structures were markedly more stable than non-acylated anthocyanins, nevertheless the after-digestion total recovery of anthocyanins was about 25%.

Feeding twelve volunteers with increasing doses (100, 200, and 300 g) of steamed red cabbage containing 1.38 μmol of anthocyanins/g (containing 30 acylated and 6 non-acylated anthocyanins), Charron et al. [253] evaluated the red cabbage anthocyanin bioavailability analyzing the excretion of intact and metabolized anthocyanin compounds in the urine. After 24 h, from the excreted urine were recovered 3 non-acylated and 8 acylated intact anthocyanins and 4 glucuronidated and methylated anthocyanin metabolites. Overall, the recovery of anthocyanins in excreted urine was four times higher for non-acylated compared to the acylated anthocyanins.

Comparing the biological activities (antioxidant, cytotoxic, anti-genotoxic, and influence on enzymatic activities) of the extract of green and red cabbage Koss-Mikołajczyk et al. [250] found that the anthocyanin content and profile was highly correlated with the antioxidant capacity of tested plant extracts measured through different spectrophotometric assays (ABTS, FC, DPPH, and FRAP), and by testing the cellular antioxidant activity. Instead, all the other biological activities tested were not correlated with the content of neither anthocyanins nor glucosinolate derivatives, suggesting that the food matrix effect may be more relevant than the biological activity of the single compound. This aspect should be further examined considering that other cabbage-like vegetables may be subject to different processing and anthocyanins and glucosinolates may have different levels of stability depending on the type of thermal or non-thermal processing and even the effect of the food matrix may change [19,254].

### 4.3. Artichoke

Native of the Mediterranean Basin and domesticated in Southern Italy during the Roman Empire [255,256], artichoke [*Cynara cardunculus* L. var. *scòlymus* (L.) Fiori] or globe artichoke constitutes a rich source of bioactive compounds and an important component of the Mediterranean diet to which are attributed a number of medicinal properties [20,21]. Artichoke is a major vegetable crop gaining popularity as a natural functional food. Grown on about 122,390 ha worldwide, over 1.5 million tons of artichoke heads were produced in 2017 [27]. As traditional producer and consumer of artichokes, Italy (33.1% of globe artichoke harvested area), Spain (13.4%), France (6.2%), and a few other European countries in the Mediterranean area continue to dominate the production of artichoke at global level. Nevertheless, their share is decreasing as the cultivation of this crop and its consumption are gradually expanding in other regions and reached important land investment in Egypt (10,159 ha), Algeria (5532 ha), Tunisia (3687 ha), Turkey (2994 ha), and Morocco (2923 ha) within the Mediterranean Basin, westward in Perú (8646 ha), Argentina (4472 ha), United States (2914 ha), and Chile (1464 ha), as well as eastward, especially in China (11,803 ha) that has the third largest investment on artichoke crops after Italy and Spain [27]. As a perennial crop, artichoke produces a robust stalk and large leaf biomass (about 75–80% of the above-ground plant biomass) from which during the reproductive phase the floral stems emerge sustaining the primary edible portion constituted by the immature inflorescences called heads or capitula. The capitula is constituted by a fleshy receptacle (the base of the inflorescence) with the inner heart made of tender bracts protected by more fibrous external bracts. The immature inflorescences are a rich source of macro- and microminerals, dietary fibers, inulin, vitamins, sesquiterpene lactones, and phenolic compounds including a series of flavonoids compounds and anthocyanins that are responsible for the pigmentation of the bracts characterizing the heads of different genotypes [20,21,257,258]. The pigmentation of the heads is in fact along with other morphology parameters (shape, presence of spines on bracts) and agronomic characteristics (earliness), one of the main factors used to classify artichoke varietal types. Based on the pigmentation of the heads, artichoke landraces and new hybrids are distinguished in two groups, namely green and pigmented artichokes. The pigmentation of the heads is considered an important quality parameter affecting consumer acceptance in different regions [20,259], and often it is anticipated in the name of the varietal type especially for the pigmented varietal type like in the case of the French “Violet de Provence”, “Violet de Hyères”, and “Violet du Gapeau”, or the Italian “Violetto di provenza”, “Violetto di Sicilia”, “Violetto Toscano”, “Violetto di S. Erasmo”, and “Violetto di Chioggia” [260]. In a recent study on the inheritance of artichoke bract pigmentation, Portis et al. [261] found a good level of heritability for bract pigmentation. However, previous studies show that there is large variations of the pigmentation of the bracts within the same landrace, and in the case of artichoke the biosynthesis and accumulation of anthocyanins is highly influenced by environmental conditions and especially by temperatures [261,262]. Artichoke pigmentation is considered a complex trait in which several metabolic pathways could be involved. In 1969, Pochard et al. [263] proposed that the anthocyanin pigmentation in artichoke could be genetically determined by one or two major genes with the involvement of several modifiers. Later, based on the segregation pattern observed in populations obtained crossing inbreed lines and clones and self-pollinated clones of different genetic origins, Cravero et al. [264] proposed that the pigmentation of artichoke heads may be controlled genetically by two independent genes P and U with a simple recessive epistasis, where plants of genotype PP or Pp allow the biosynthesis of anthocyanins and result in purple bracts whereas pp genotype inhibits anthocyanin synthesis resulting in green bracts; at the same time genotypes UU or Uu are characterized by a non-uniform pigmentation encoded by the allele P, and uu genotypes develop uniformly pigmented bracts in presence of the allele P. While further studies should confirm this simple model, the variability of the pigmentation observed within the same genotype suggests that the genetic basis for anthocyanin pigmentation of artichoke bracts is more complex and likely the two loci model proposed is regulated by several modifier genes or multiple alleles that determine the expression of the intensity of the pigmentation in artichoke heads [261,262,264]. More recently, De Palma et al. [265] isolated and functionally characterized CcF3’H as the first structural gene of the flavonoid biosynthesis (encoding for flavonoid 3’-hydroxylase) from *C. cardunculus.* While Blanco et al. [266] isolated and functionally characterized CcMYB12, the artichoke putative homologue of the transcription factor R2R3-MYBs expressing proteins that regulate the biosynthesis of flavonoids and anthocyanins in many species.

Analyzing the polyphenolic profile of artichokes in different genotypes, it is possible to identify two main classes of phenolic compounds, namely hydroxicinnamic acids (C3-C6 skeleton) and flavonoids (C6-C3-C6 skeleton). Within the first class chlorogenic, 3,5-*O*-dicaffeoylquinic, and 1,5-*O*-dicaffeoylquinic acids are the predominant compounds followed by other minor mono- and di-caffeoylquinic acids [20,21,267]. Among the flavonoids that quantitatively represent about 10% of the total phenolic compound in artichoke [20], the flavones apigenin-7-*O*-glucuronide, apigenin-7-*O*-rutinoside, apigenin-7-*O*-glucoside, luteolin-7-*O*-glucuronide, luteolin-7-*O*-rutinoside, luteolin-7-*O*-glucoside, naringenin-7-*O*-rutinoside, and naringenin-7-*O*-glucoside are the predominant compounds, followed by the anthocyanins including cyanidin, peonidin, and delphinidin derivatives [20,21,257,267,268]. Other studies have revealed that specific phenolic compounds are accumulated only in certain genotypes and certain plant portions even within the same capitula [269,270].

The first attempts to identify the anthocyanin profile of green and purple artichokes were conducted in the late 1970s [20]. However, only later Schütz et al. [271] analyzing by HPLC the pigmented bracts of seven different varietal types of artichoke isolated and identified the main anthocyanins as cyanidin 3,5-diglucoside, cyanidin 3-glucoside, cyanidin 3,5-malonyldiglucoside, cyanidin 3-(3′′-malonyl)glucoside, and cyanidin 3-(6′′-malonyl)glucoside along with some minor compounds such as peonidin 3-glucoside, peonidin 3-(6′′-malonyl)glucoside, and delphinidin glycoside. The same authors observed that the anthocyanin profile varies between different genotypes, nevertheless limited information is available on the genotypic variation of these compounds in artichoke. Analyzing new hybrids and local landraces characterized by bracts with different levels of pigmentation, Bonasia et al. [272] observed that “Violetto di Provenza” and “Tempo” characterized by higher pigmentation had also the highest total phenolic content in the heart and external bracts and in the external bracts, respectively. Examining the content of total anthocyanins in bracts, leaves, and floral stems of two genotypes grown in Tunisia, namely “Violet d’Hyéres” and “Blanc d’Oran”, Dabbou et al. [273] found that leaves of “Blanc d’Oran” had the highest concentration of anthocyanins (20.5 µg/g DW) while bracts and floral stems had the lowest concentration (8.3 and 5.9 µg/g DW, respectively). Instead, lower variability between different plant portions was observed in the case of “Violet d’Hyéres” that on average had a total anthocyanin concentration of 14.2 µg/g DW). These results suggest that even the leaves of artichoke plants which represent a big portion of plant biomass may constitute a good source of anthocyanins besides being a source of other phenolic compounds [274]. A very limited number of studies have been conducted to evaluate specifically the biological activity of artichoke anthocyanins [257]. The main bioactive effect attributed to anthocyanins, flavonoids, and other phenolic compounds extracted from artichoke plant tissues is their antioxidant activity demonstrated by several in vitro and in vivo studies [20,21,275,276]. Anthocyanins have also shown lipid lowering effects in a placebo-control double-blind study by reducing serum LDL cholesterol by 7.9%, triglycerides by 23.0%, apolipoprotein by 16.5%, and apolipoprotein C-III by 11.0%, and increasing HDL cholesterol by 19.4% compared with placebo after administration of 160 mg of anthocyanins for 24 weeks twice daily [277]. Intake of anthocyanins seems to have positive effects on the cardiovascular system by also reducing arterial stiffness [278]. In another study, it was observed that artichoke leaf extracts and artichoke flavonoids up-regulate the gene expression of endothelial-type nitric oxide synthase (eNOS, a vasoprotective molecule) in human endothelial cells [279]. While in a follow-up study Xia et al. [280] observed that treatment of human coronary artery smooth muscle cells (HCASMC) with artichoke leaf extracts and particularly with cynarin and cyanidin induced a down-regulation of inducible nitrous oxide synthase (iNOS, a pro-inflammatory molecule that can cause vascular dysfunctions), suggesting that artichoke flavonoid compounds may have great therapeutic potential. In recent years, a number of studies contributed to demonstrate that artichoke heads and leaf extracts and, in some cases, specific phenolic compounds have health-beneficial properties including anti-inflammatory activity, anti-bacterial activity, hepatoprotective activity, hypocholesterolemic and low density lipoproteins (LDL) oxidation inhibition effect, hypoglycemic effect, as well as anticancer activity [20,21,257]. Nevertheless, in most of the cases these biological activities cannot be attributed to a single compound, but are determined by the combined synergistic effect of different compounds [20,21]. In this perspective, the matrix effect is fundamental in determining the bioaccessibility, bioavailability, and the effect of polyphenols, especially considering that polyphenols and anthocyanins in particular have a relatively low bioavailability being quickly transformed into derivatives of phenolic acids [21,281,282,283].

### 4.4. Asparagus

Used since ancient time for the diuretic and medicinal properties of its spears [284], cultivated asparagus (*Asparagus officinalis* L.) is now a popular vegetable grown worldwide on over 1.5 million hectares producing over 8.9 million tons of spears [27]. Native of Eastern Europe, cultivated asparagus is grown at commercial level mainly in China, followed far behind by Perú, Mexico, Germany, Spain, USA, Italy, Japan, France, and the Netherlands. The edible portion of this herbaceous perennial is constituted by its spears or young stems produced by the underground crown, which may be green, green-purple, or purple when harvested above ground or white when purposely harvested before exposed to sunlight. Asparagus spears are considered a rich source of minerals [285], amino acids and dietary fibers [286], saponins [287,288], vitamins and volatile sulfur organic compounds [289], and especially of phenolic compounds and flavonoids [290], including anthocyanins that are responsible for the purple color of the bracts of green spears or of the whole spears in purple asparagus genotypes [23,154,291]. Being rich of all these bioactive compounds it is difficult to isolate the effect of anthocyanins and specific pigments, nevertheless several studies have demonstrated the antioxidant properties of anthocyanins in the species [284]. Comparing green, white, and purple spears, Maeda et al. [291] found that purple spears had significantly higher levels of rutin compared to green spears, while rutin was not detected in white asparagus spears. The same authors observed a positive correlation between spears total polyphenol content, rutin, and DPPH radical scavenging activities, suggesting that purple asparagus may provide higher levels of antioxidants compared to green and white asparagus [291]. In this perspective, while most of the cultivars have been selected to produce green spears and have a relatively small content of anthocyanins, and even less pigments when etiolated to produce white spears, more recently, as for other crops, specific functional breeding programs have developed purple cultivars characterized by high levels of anthocyanins to satisfy the need of consumers attracted by new colors and seeking richer sources of natural antioxidants [291]. Most of the modern commercial cultivars of green and white asparagus are diploid (2*n* = 20) and were developed from an old population called “Purple Dutch” from which French growers selected two stocks called “Precoce d’Argenteuil” and “Tardive d’Argenteuil” which were subsequently used to develop modern cultivars and hybrids in The Netherlands, France, Germany, United Kingdom, and in the USA [292,293]. Although any green asparagus genotype may be used to produce etiolated white spears, Dutch, French, and German breeding programs have selected cultivars suitable specifically for white spears production to satisfy the European market requirements by reducing as much possible the content of anthocyanins that may develop and accumulate in the tips of white-harvested spears even during storage [292,294]. On the other hand, using “Violetto d’Albenga” a tetraploid local population traditionally grown in Northern Italy, likely derived from interspecific crossing between *A. officinalis* and *A. maritimus* [295] and characterized by the production of larger, sweeter, and less fibrous spears ranging in color from green to dark purple, breeding programs in the US and New Zealand have developed in the early 1990s the first cultivars producing purple spears such as “Purple Passion” [296] and “Purple Pacific” and “Stewarts Purple” [297].

Using HPLC and NMR to analyze the fresh peel of “Purple Passion” asparagus, Sakaguchi et al. [23] isolated two major anthocyanins identified as: cyanidin 3-[3’’-(*O*-β-d-glucopyranosyl)-6’’-(*O*-α-l-rhamnopyranosyl)-*O*-β-d-glucopyranoside], and cyanidin 3-rutinoside characterized by high antioxidant activity as determined through ORAC (Oxygen Radical Absorbance Capacity) assay. Recently, Dong et al. [298] comparing the anthocyanin profile of three purple asparagus cultivars (“Jing Zi-2”, “Purple Passion”, and “Pacific Purple”) with a green control (“Jing Lv-1”), identified sixteen anthocyanins, with peonidin, cyanidin and their glycoside derivatives being the predominant compounds. In the same study, through transcriptomics and quantitative real-time polymerase chain reaction (qRT-PCR) analysis, several anthocyanin synthesizing genes (PAL, C4h, 4CL, CHS, CHI, F3H, F3’H, DFR, ANS, and 3GT) and transcription factors genes (bHLH137-like, TT2-like, WD40-like, bZIP61-like, and MADS18-like) that regulates the biosynthesis of anthocyanins like reported for other species were identified and showed to be differentially expressed between purple and green cultivars [298]. The expression of the same genes and the content of anthocyanins were examined also comparing spears grown in the presence of light and in the dark. In accordance with previous studies [299,300], results demonstrated that anthocyanin biosynthetic and regulatory genes are considerably down-regulated in absence of light and pigments are not synthesized in dark conditions, suggesting that light conditions are a key factor for anthocyanin biosynthesis and accumulation. This is further corroborated by the findings of Huyskens-Keil et al. [301] who showed the effects of light quality (white, red, blue, UV-C) on the activity of phenylalanine ammonia-lyase (PAL) and peroxidase (POD) and the synthesis and accumulation of anthocyanins in basal and apical segments of white asparagus spears during postharvest storage. Postharvest conditions and handling may in fact considerably affect the stability of anthocyanins. In another recent study, Barberis et al. [302] reported that dipping the spears of “Purple Passion” for 5 min in a 3 mM solution of oxalic acid (pH 2.9) reduced the lightness of the spears after 12 days only by 13% compared to spears treated with water (control pH 8) or with 1 mM oxalic acid (pH 6) solution, suggesting that the low pH treatment enhanced the stability of the anthocyanin pigments. Analyzing the phenolic profile of green and purple asparagus at harvest and during storage, in accordance with Sakaguchi et al. [23] the same authors [302] found that cyanidin glucosyl rutinoside, cyanidin rutinoside and peonidin rutinoside (at harvest 774.2, 125.5, and 84.8 mg/kg, respectively) were the main anthocyanins identified in “Purple Passion” spears and determining the typical purple color. During storage, the first anthocyanin decreased overtime and was negatively affected by oxalic acid treatment, cyanidin rutinoside increased over time, while peonidin rutinoside remained stable.

### 4.5. Sweet Corn

Native to America, corn (*Zea mays* L.) has been cultivated in Central America by indigenous populations at least since 3500 BC [303]. When the European explorers arrived in North America, Iroquois Indians living in the region corresponding to the current state of Pennsylvania and New York grew a variety of sweet corn (*Zea mays* L. *saccharata* Sturt.) that turned blue upon ripening, along with other multi-colored varieties [304]. The first samples of maize seeds that arrived in Spain were described as characterized by different colors ranging from white to black [305,306]. Initially grown for curiosity, with the arrival of seeds from North America adapted to higher latitudes, and thus more suitable to the European photoperiod and climate conditions, the new cereal crop started to spread from Spain to the rest of Europe. The rapid diffusion of maize in Europe is also testified by the painter Giuseppe Arcimboldo, in his famous painting “Summer” made in 1573 in which a maize ear is visible [305]. With the process of selection initially conducted in Europe the new crop lost its multi-color pigmentation with the exceptions of a few local populations and assumed the typical white or yellow color of the varieties currently cultivated worldwide.

With over 1.134 million metric tons of corn produced at global level in 2017 [27], corn is by far the first cereal produced in the world, second in terms of acreage only to wheat. Among the different types of maize, sweet corn is particularly important as it is used for human consumption worldwide. In the US, first producer of sweet corn, in 2017 were harvested 464,600 acres (about 188.000 ha) of sweet corn generating a crop value of over $892 million [307]. About 75% of the sweet corn is produced for the fresh market and the remaining portion is used for canned and frozen food processing, making sweet corn the second most important processing vegetable after processing tomato.

Sweet corn is derived from a natural genetic mutation of field corn (dent corn) that was first reported in Pennsylvania in 1770, although it was probably cultivated before by native Americans [308]. The mutant genotype *sugary* (*su*) accumulates more sugar in the endosperm (10.2%) than the standard starchy maize (3.5%) resulting in sweeter taste. Years later, several sugary sweet corn varieties have been selected. These traditional varieties characterized mainly by white or yellow kernels, are harvested before complete physiological ripening when the level of sugar is maximum and have short shelf-life because after harvest the sugar is rapidly converted to starch [309]. Subsequently, sweeter hybrid varieties have been developed by selecting *sugar-enhanced* (*se*) mutants characterized by double content of sugar (20–35%), tender kernels, creamy texture and good corn flavor. Yet, more recently *supersweet* varieties have been developed selecting *shrunken-2* (*sh2*) mutants characterized by even higher sugar content at the immature milky stage (29.9%) and extended shelf life, with lighter shrunken kernels. Lately high sugar hybrids are developed combining *su*, *se*, and *sh2* genes to obtain optimal combinations of sugar, texture, taste, and long shelf life [309,310]. Most of the sweet corn hybrids grown and consumed today at commercial level are characterized by yellow (60%), white (20%), or bicolor (20%, yellow and white) kernels. Nevertheless, in recent years, the interest in reviving ancient colored sweet corn varieties or developing new pigmented varieties characterized by high content of carotenoids [311] and especially anthocyanins is increasing [305,312,313,314,315,316] due to the potential functional properties of anthocyanin-rich genotypes [317,318], as well as to the increasing demand of natural colorants [22,319,320]. The biosynthesis of anthocyanins in maize aleurone (external part of the endosperm) and pericarp (external part of the kernel) of the kernels or in plant tissues involve over twenty structural and regulatory genes that have been identified and functionally characterized as reviewed by Petroni et al. [305,321,322,323]. The wide range of colors observed in maize kernels is mainly due to the biosynthesis and accumulation of carotenoids and flavonoids. Carotenoids such as β-carotene, zeaxanthin and lutein which are lipid-soluble pigments and are responsible for the color of kernels ranging from yellow to deep orange [311,324]. Maize flavonoids include two main classes of pigments: (i) phlobaphenes which are water-insoluble 3-deoxyflavanoid pigments that accumulate in the pericarp of the kernels and the cob and are responsible for the development of kernel colors ranging from orange to brick red [315]; and (ii) anthocyanins which are water-soluble pigments responsible for the development of pink, red, purple, and blue color in the aleurone and pericarp of the kernels as well as in other plant tissues. Within the anthocyanins, the main class of pigments identified are cyanidin, peonidin, and pelargonidin derivatives with the first two providing bluish-red color and the latter being responsible for more orange-red color [22]. The combination of carotenoids and flavonoids in pericarp and aleurone generates an incredible variety of shades [22] and may be influenced also by the condensation with flavanols [319,320,325,326] or other modifications of the anthocyanin compounds. Analyzing the anthocyanin profile of the kernels of six different purple maize genotypes, González-Manzano et al. [326] identified the dimer catechin-(4a-8)-cyanidin-3,5-*O*-diglucoside and other flavanol-anthocyanin condensed pigments along with several related anthocyanin pigments such as cyanidin-3-glucoside, cyanidin-3-malonylglucoside, peonidin-3-glucoside, peonidin-3-malonylglucoside, pelargonidin-3-dimalonylglucoside, and other derivatives. Malonyl- and dimalonyl acylated anthocyanins are particularly interesting for their higher stability as natural colorants [22]. Analyzing the extract of corn cobs and kernels of a Chinese purple corn, Yang and Zhai [327] identified cyanidin-3-glucoside, pelargonidin-3-glucoside and peonidin-3-glucoside including their malonated derivatives. Besides the great potential of maize pigments as natural food colorants [22,319], there is great interest in the development of anthocyanin-rich maize functional food products [305,311,312,321,328]. Several studies have reported the potential health-beneficial properties of anthocyanin-rich maize [305,317,329]. Examining the phenolic and anthocyanin content of eighteen Mexican maize genotypes, Lopez-Martinez et al. found that total anthocyanins ranged from 1.54 to 850.9 mg of cyanidin-glucoside equivalents/100 g of whole grain flour. Purple genotypes rich in anthocyanins exhibited also the greatest antioxidant activity [330]. Andean purple corn had higher antioxidant activity and antiradical kinetics than blueberries and higher or similar level of anthocyanins [331]. Similarly, analyzing 49 lines of waxy corn (*Zea mays* L. var. *ceratina*) characterized by different colors Harakotr et al. [332] found a large variability of phenolic and anthocyanin compounds and a positive correlation coefficient between anthocyanins and 2,2-diphenyl-1-picrylhydrazyl (DPPH) radical scavenging ability (*r* = 0.94) and between anthocyanins and Trolox equivalent antioxidant capacity (TEAC, *r* = 0.88). Introduction in the diet of anthocyanin-rich corn may contribute to prevent also obesity and diabetes. Tsuda et al. [317] found that feeding high fat (HF) mice for 12 weeks with cyanidin 3-glucoside-rich purple corn significantly reduced HF body weight and white and brown adipose tissues compared to HF mice not receiving purple corn supplement. The HF diet induced also hyperglycemia, hyperinsulinemia, and hyperleptinemia, whereas the same physiological conditions were not observed in HF mice receiving the purple corn supplement. Moreover, an increase of tumor necrosis factor (TNF)-α mRNA level was observed in the HF-diet while it was not observed in the HF group receiving the purple corn supplement which suppressed the mRNA of enzymes involved in fatty acid and triacylglycerol synthesis and reduced the level of sterol regulatory element binding protein-1 mRNA in white adipose tissue. Using near-isogenic maize lines which differed only in the presence or not of anthocyanins, and feeding rats with anthocyanin-rich and anthocyanin-deprived maize for 8 weeks it was observed that in the group fed with anthocyanin-rich corn cardiac tissue damaged following ischemic conditions was reduced by about 30% compared to the group fed with anthocyanin-deprived corn [333]. An increase of myocardial glutathione and omega-3 fatty acids levels in blood indicated that diet supplementation with anthocyanins regulated the cardiac antioxidant defense and the conversion of α-linolenic acid to omega-3 fatty acids [333,334]. In an in vivo study, using db/db mice fed with 10 mg/kg of purple corn extract for 8 weeks, Kang et al. [335] found that anthocyanin-rich purple corn extracts reduced glomerular angiogenesis of diabetic kidneys by inhibiting the induction of vascular endothelial growth factor (VEGF) and hypoxia inducible factor (HIF)-1a induced by hyperglycemic condition. Kang et al. results demonstrated that purple corn extract inhibited glomerular angiogenesis caused by chronic hyperglycemia and diabetes by disturbing the Angpt-Tie-2 ligand-receptor system linked to the renal VEGF receptor 2 (VEGFR2), suggesting that purple corn extract could be used to target abnormal angiogenesis in diabetic nephropathy leading to kidney failure [335]. Yet, in a recent study Mazewski et al. [336] found that anthocyanin-rich purple and red corn may potentially contribute to inhibiting human colon cancer cell proliferation by promoting apoptosis and suppressing angiogenesis.

The main pigments isolated in vegetables consumed for various plant parts are presented in Table 3.

## 5. Conclusions

Vegetable products are pivotal in human health and their daily consumption is highly recommended due to their high content in phytochemicals with diverse beneficial health effects. The vegetable crops presented in this review, namely leafy and fruit vegetables and species where other plant parts are consumed, are well known and widely consumed throughout the world, although their colored variants are not always accepted by consumers and proper marketing is needed to introduce them to the public. Currently, the food and beverage industry is seeking for natural alternatives of synthetic compounds to satiate market demands for “non-chemical additives” food products. Therefore, the broad color palette the presented vegetables exhibit not only could diversify human diet and make food products more attractive to consumers and increase their appeal, but also could be proved as valuable sources of natural coloring agents. Under the circumstances, future research should focus on the identification of those promising coloring agents and the establishment of efficient and sustainable techniques for their isolation. Other future requirements, include breeding and agronomic protocols that could improve pigments content in the final produce, as well as post-harvest and processing conditions to increase extraction efficiency of natural coloring agents. Finally, the mechanisms of actions related with the health effects and antioxidant activities of coloring compounds have to be revealed and valorized in the pharmaceutical and food industry for the design of novel functional foods and drugs.

## Figures and Tables

**Table 1:** The main pigments isolated in various leafy vegetables.

Species	Color	Class of Compounds	Compounds (Content on a Fresh Weight (fw) or Dry Weight (dw) Basis)	References
Lettuce (*Lactuca sativa* L.)	Red	Anthocyanins	cyanidin (1558.0–3656.9 μγ/g dw), cyanidin-3-*O*-(6’’-malonyl-β-glucopyranoside), cyanidin-3-*O*-(6’’-malonyl-β-glucopyranoside methyl ester), cyanidin-3-*O*-β-glucopyranoside, cyanidin-3-glucoside (1.40–3.07 g/100 g fw)	[42,43,46,48]
		Carotenoids	all-*E*-violaxanthin (23.9–33.4 μg/g fw), 9′-*Z*-neoxanthin (11.3–14.6 μg/g fw), all-*E*-Luteoxanthin, all-*E*-lactucaxanthin (19.7–23.0 μg/g fw), all-*E*-lutein (31.3–38.2 μg/g fw), and all-*E*-β-carotene (9.0–13.3 μg/g fw)	[42]
	Green	Carotenoids	all-*E*-violaxanthin (15.9–37.1 μg/g fw), 9′-*Z*-neoxanthin (5.0–11.4 μg/g fw), all-*E*-Luteoxanthin, all-*E*-lactucaxanthin (7.5–17.4 μg/g fw), all-*E*-lutein (16.4–36.3 μg/g fw), and all-*E*-β-carotene (4.2–12.9 μg/g fw)	[42]
		Chlorophylls	Chlorophyll a and b (6.95–26.92 mg/100 g fw and 4.60–10.30 mg/100 g fw, respectively)	[43,48]
		Anthocyanins	Cyanidin-3-glucoside (0.192–0.260 g/100 g fw)	[48]
Basil (*Ocimum basilicum* L.).	Purple/red	Anthocyanins	Anthocyanin A (0.325–0.423 mg/g dw),-anthocyanin B (0.057–0.641 mg/g dw), anthocyanin C (0.362–0.877 mg/g dw), anthocyanin D (0.063–0.662 mg/g dw), cyanidin-based (1.78–3.18 mg/g dw) pigments- and peonidin-based (19.8% fw) pigments, cyanidin-3-(6,6′-di-p-coumaroyl)-sophoroside-5-glcucoside (7.5 mg/g extract)	[63,68,115,116]
	Green	Carotenoids	Lutein (4.99–6.64 mg/100 g fw or 22.1–24.0 mg/100 g dw), β-carotene 4.42–6.01 mg/100 g fw, zeaxanthin (0.30–0.45 mg/100 g fw or 1.8 mg/100 g dw)	[62,64]
Perilla (*Perilla frutescens* L. Briit)	Red	Anthocyanins	Cyanidin and cyanidin derivatives (6.44 mg/g dw), shisonin (0.126–0.416 mg/g dw), malonylshishonin (0.462–1.116 mg/g dw)	[83,117,118,119,120]
Swiss chard (*Beta vulgaris* var. *cicla* L.)	Yellow	Betaxanthins	Vulgaxanthin I, miraxanthin V	[121,122]
	Red/purple	Betacyanins	Betanin, isobetanin, betanidin, and isobetanidin	[123]

**Table 2:** The main pigments isolated in various fruit vegetables.

Species	Color	Class of Compounds	Compounds	References
Tomato *Solanum lycopersicum* L.	Red tomato	Carotenoids	Lycopene, phytoene, phytofluene, ζ-carotene, γ-carotene, β-carotene, neurosporene, lutein	[127,149]
	Purple tomato	Carotenoids	Lycopene (36.51–61.30 mg/kg fw), β-carotene (6.5–6.8 mg/kg fw), lutein	[128,136]
	Purple tomato	Petunidin and malvidin acylglycosides	petunidin-3-*O*-caffeoyl-rutinoside-5-*O*-glucoside (1.88–15.36 mg/100 g dw), petunidin-3-*O*-(*p*-coumaryl)-rutinoside-5-O-glucoside (16.97–50.18 mg/100 g dw)1, malvidin-3-*O*-(*p*-coumaryl)-rutinoside-5-*O*-glucoside (6.17-27.06 mg/100 g dw), delphinidin-3-(trans-coumaroyl)-rutinoside-5-glucoside (114.53–162.43 mg/100 g dw)	[7,136,156]
	Purple/black tomato	Petunidin and malvidin acylglycosides	petunidin 3-(6-(4-(*E*-*p*-coumaroyl)rhamnosyl)glucoside)-5-glucoside (petanin) (2.77 mg/g dw), Malvidin 3-(6-(4-(*E*-*p*-coumaroyl)rhamnosyl)glucoside)-5-glucoside (1.05 mg/g dw)	[130]
Eggplant *Solanum melongena* L.	Purple eggplant	delphinidin derivatives	delphinidin-3 glucoside-5-(coumaryl) dirhamnoside (1.10 mg/g dw), delphinidin-3-(*p*-coumaroylrutinoside)-5-glucoside (1357–3200 mg/kg dw)	[173,186,187,223]
Pepper *Capsicum* spp. L.	Green/red peppers	Carotenoids	β-carotene, lycopene, zeaxanthin	[194]
	Purple/black pepper	Delphinidin, cyanidin and malvidin derivatives	delphinidin 3,5-diglucoside, delphinidin 3-*O*-rutinoside, delphinidin-3-*trans*-coumaroylrutinoside-5-glucoside (284.8 μg/g fw), delphinidin-3-*cis* coumaroylrutinoside-5-glucoside (14.72 μg/g fw)	[181,182,200]
Lablab *Lablab purpureus* L.	purple lablab	Malvidin and petunidin derivatives	malvidin 3-sambubiose, malvidin 3-glucoside, delphinidin 3-glucoside-5-rutinoside, delphinidin 3-glucose-5-rhamnose and petunidin 3-rutinose	[213]
Common bean *Phaseolus vulgaris* L.	Black bean	Pelargonidin, Cyaniding and delphinidin glucosides	pelargonidin-3-glucoside, cyanidin-3-glucoside, delphinidin-3-glucoside	[217]

**Table 3:** The main pigments isolated in vegetables consumed for plant parts other than fruit and leaves.

Species	Edible Part	Color	Class of Compounds	Compounds	References
Broccoli (*Brassica oleracea* L., var. *italica* Plenk)	Inflorescence	Purple	Acylated anthocyanins	cyanidin 3-*O*-(sinapoyl)(sinapoyl)diglucoside-5-*O*-glucoside (0.0653–0.3716 mg/100 g fw), cyanidin 3-*O*-(sinapoyl)diglucoside-5-*O*-glucoside (0.0119–0.0158 mg/100 g fw), cyanidin 3-*O*-(feruloyl)diglucoside-5-*O*-glucoside (0.0201–0.0765 mg/100 g fw), cyanidin 3-*O*-(sinapoyl)(feruloyl)diglucoside-5-*O*-(malonyl)glucoside (0.013–0.048 mg/100 g fw), and cyanidin 3-*O*-(sinapoyl)(sinapoyl)diglucoside-5-*O*-(malonyl)glucoside (0.0159–0.1035 mg/100 g fw)	[231,233]
Cauliflower (*Brassica oleracea* L., var. *botrytis*)	Inflorescence	Purple or dark violet	Cyanidin derivatives	cyanidin-3-(6-*p*-coumaryl)-sophoroside-5-glucoside (9.0–29.9 mg/kg fw), cyanidin-3-(6-*p*-coumaryl)-sophoroside-5-(6-sinapyl)-glucoside (27.8–37.8 mg/kg fw)	[19]
Cabbage (*Brassica oleracea* L. var. *capitata*)	Leaves	Purple	Cyanidin derivatives	cyanidin-3-diglucoside-5-glucoside (0.64 mg/g dw), cyanidin-3-(sinapoyl)(sinapoyl)-diglucoside-5-glucoside (0.26-0.58 mg/g dw), cyanidin-3-(sinapoyl)-diglucoside-5-glucoside (0.85 mg/g dw)and cyanidin-3-(*p*-coumaroyl)-diglucoside-5-glucoside (0.19-0.92 mg/g dw), cyanidin-3-(feruloyl)-diglucoside-5-glucoside (0.14–0.55 mg/g dw)	[249,250,252]
Artichoke [*Cynara cardunculus* L. var. *scòlymus* (L.) Fiori]	Immature inflorescence	Purple	Cyanidin, peonidin, and delphinidin derivatives	cyanidin 3,5-diglucoside (0–11.7 mg/kg dw), cyanidin 3-glucoside (0.3–194.1 mg/kg dw), cyanidin 3,5-malonyldiglucoside (0.5-218.0 mg/kg dw), cyanidin 3-(3′′-malonyl)glucoside (0–47.3 mg/kg dw), and cyanidin 3-(6′′-malonyl)glucoside (7.6-1234.3 mg/kg dw) along with some minor compounds such as peonidin 3-glucoside, peonidin 3-(6′′-malonyl)glucoside, and delphinidin glycoside.	[271]
Asparagus (*Asparagus officinalis* L.)	Spears (young stems)	Purple	Cyanidin and peonidin derivatives	Cyanidin 3-[3’’-(*O*-β-d-glucopyranosyl)-6’’-(*O*-α-l-rhamnopyranosyl)-*O*-β-d-glucopyranoside], cyanidin 3-rutinoside (774.2 mg/kg fw), cyanidin glucosyl rutinoside (125.5 mg/kg fw), peonidin rutinoside (84.8 mg/kg fw)	[23,298,302]
Sweet corn (*Zea mays saccharata* Sturt.)	Maize kernels	Yellow – deep orange	Carotenoids	β-carotene (2.62 μg/g dw), β-cryptoxanthin (3.96 μg/g dw), zeaxanthin (23.74 μg/g fw), zeinoxanthin (0.75 μg/g dw), lutein (8.2 μg/g dw), antheraxanthin (2.5 μg/g dw))	[311,324]
	Maize kernel pericarp and cobs	Orange – red brik	Phlobaphenes	Phlobaphenes (320.24 A_510_/100 g)	[315]
	Maize cobs and kernel aleurone and pericarp	Pink, red, purple, blue	Anthocyanins (cyanidin, penodin, pelargonidin,	cyanidin-3-glucoside, pelargonidin-3- glucoside, penodin-3-glucoside, cyanidin-3-(6-malonylglucoside), pelargonidin-3-(6-malonylglucoside), peonidin-3-(6-malonylglucoside), peonidin-3-glucoside, peonidin-3-malonylglucoside, pelargonidin-3-dimalonylglucoside	[326,327]
